# Efficacy of autologous mesenchymal stromal cell treatment for chronic degenerative musculoskeletal conditions in dogs: A retrospective study

**DOI:** 10.3389/fvets.2022.1014687

**Published:** 2023-01-13

**Authors:** Andrew J. Armitage, Joanna M. Miller, Tim H. Sparks, Alex E. Georgiou, Jacqueline Reid

**Affiliations:** ^1^Greenside Veterinary Practice, Part of Linnaeus Veterinary Limited, Melrose, United Kingdom; ^2^Cell Therapy Sciences Ltd., Coventry, United Kingdom; ^3^Waltham Petcare Science Institute, Melton Mowbray, United Kingdom; ^4^Coventry University, Coventry, United Kingdom; ^5^University of Glasgow, Glasgow, United Kingdom; ^6^NewMetrica Research Ltd., Glasgow, United Kingdom

**Keywords:** canine, tendinopathies, regenerative medicine, osteoarthritis, platelet rich plasma (PRP), laser therapy, lumbosacral disease, stem cells

## Abstract

**Introduction:**

The objective of this study was to retrospectively analyze clinical data from a referral regenerative medicine practice, to investigate the efficacy of autologous mesenchymal stromal cells (MSC) in 245 dogs deemed unresponsive to conventional treatment by their referring vet.

**Methods:**

Diagnostic imaging [radiology and musculoskeletal ultrasound (MSK-US)] identified musculoskeletal pathology holistically. MSCs, produced according to current guidelines, were initially administered with PRP by targeted injection to joints and/or tendons, with a second MSC monotherapy administered 12 weeks later to dogs with severe pathology and/or previous elbow arthroscopic interventions. Dogs with lumbosacral disease received epidural MSCs with additional intravenous MSCs administered to dogs with spondylosis of the cervical, thoracic and lumbar spine. All dogs received laser therapy at 10 J/cm^2^ at the time of treatment and for 5 sessions thereafter. Objective outcome measures (stance analysis, range of joint motion, pressure algometry) and validated subjective outcome measures (owner reported VetMetrica HRQL™ and veterinary pain and quality of life impact scores) were used to investigate short and long-term (6–104 weeks) efficacy. Outcome data were collected at predetermined time windows (0–6, 7–12, 13–18, 19–24, 25–48, 49–78, 79–104) weeks after initial treatment.

**Results:**

There were statistically significant improvements in post compared with pre-treatment measures at all time windows in stance analysis, shoulder and hip range of motion, lumbosacral pressure algometry, and to 49–78 weeks in carpus and elbow range of motion. Improvements in 4 domains of quality of life as measured by VetMetricaTM were statistically significant, as were scores in vet-assessed pain and quality of life impact. In dogs receiving one initial treatment the mean time before a second treatment was required to maintain improvements in objective measures was 451 days. Diagnostic imaging confirmed the regenerative effects of MSCs in tendinopathies by demonstrating resolution of abnormal mineralization and restoration of normal fiber patterns.

**Discussion:**

This represents the first study using “real-world” data to show that cell-based therapies, injected into multiple areas of musculoskeletal pathology in a targeted holistic approach, resulted in rapid and profound positive effects on the patient's pain state and quality of life which was maintained with repeat treatment for up to 2 years.

## 1. Introduction

Chronic degenerative musculoskeletal (MSK) conditions such as osteoarthritis (OA) cause significant morbidity in working and pet dogs in the UK (1, 2). Osteoarthritis, which affects 2.5% of the UK dog population is one of the most common causes of chronic pain in dogs (3). Although OA is predominately a disease of the joint, it frequently results in associated soft tissue pathology in the joint capsule and support structures as the disease progresses (4). Joint changes lead to pain and debility causing offloading of the affected joint with compensatory overloading in other areas of the locomotor apparatus and spine (5–10). There is, however, surprisingly little published evidence linking altered biomechanics to concurrent musculoskeletal diseases (MSD), but the prevalence of multiple MSD in a patient is common (11). Although there are many primary degenerative conditions that affect the MSK system, secondary changes frequently complicate the clinical picture. Many patients present with multifactorial pathologies involving various tissue types, which makes these cases challenging to treat, requiring a multimodal approach, and can lead to treatment failures when management of one condition does not apply to another. Appropriate management of MSD requires accurate diagnosis to fully evaluate the condition and a holistic approach to treatment and, as such, a single therapeutic option for multiple MSD would be beneficial in human and veterinary medicine.

The aims of treatment are to reduce pain, decrease lameness and significantly improve the patient's quality of life (QOL). Surgical options, including full or partial joint replacements, joint arthrodesis, arthroscopic interventions, and tenotomies, aimed at improving limb function and reducing pain, tend to be non-curative salvage procedures that have potentially serious complications, especially in the case of elbow dysplasia (12–16). Additional treatment options, including analgesic drugs, anti-inflammatory and monoclonal antibody medications as well as nutritional supplements, have limited efficacy, can cause side effects and are not curative (17). In contrast, regenerative medicine (RM) utilizing mesenchymal stromal cells (MSCs), which have the capacity to self-renew and differentiate into multiple cell types, has increasingly emerged as an effective clinical treatment for MSD in both human and veterinary patients (18–22). In addition to their reparative potential, they possess anti-inflammatory and immune-modulating properties that allow them to control inflammation and pain (23–27). Furthermore, several canine RM studies have included platelet rich plasma (PRP) with MSCs, as the combination is considered to be synergistic in terms of regenerative effects (22, 28).

Veterinary clinical studies using intra-articular treatments of MSCs in osteoarthritic joints have demonstrated positive and effective outcomes in terms of reduced lameness and pain, and have been shown to be safe, see Voga et al. for a comprehensive review (19). However, long-term follow up, including diagnostic imaging of their disease modifying potential, is lacking in human and veterinary medicine (19).

Outcome measures for orthopedic studies include objective measures such as force plate and kinematic gait analysis, measures of weight distribution (stance analysis), range of joint motion (ROM) and pressure algometry in cases of lumbosacral disease (LSD). While force plate and gait analysis are most frequently used, a recent publication concluded that studies utilizing a weight distribution platform (stance analysis) to monitor response to treatment in dogs with orthopedic disease would be “clinically valuable and useful for establishing research standards” (29). Similarly, goniometry has been suggested as a suitable technique for objective outcome assessment in orthopedic studies (30, 31). Pressure algometry (PA), or mechanical nociceptive testing (MNT), is an objective measure to quantify nociceptive thresholds and its use in animals has been described in a number of publications (32–37). Lane and Hill evaluated PA for measuring muscular pain at the thoracolumbar junction in dogs (36). They concluded that there was a positive increase in MNT, which related to improved muscular comfort in this region, over time in the two treatment groups but not in the control group. They propose that PA is a valid measure of MNT in the lumbar region in dogs and serves as an objective measure of muscular pain. Although clinically relevant change in PA readings has not been defined for specific anatomical locations in dogs, any increase following treatment would suggest a reduction in pain (38) which would be clinically significant even if that change was small. Various studies have shown increases in pressure pain threshold are associated with improved pain scores using clinical metrology instruments in humans with chronic lower back pain when compared in placebo-controlled trials (39, 40).

In addition to objective outcome measures which are useful for diagnosing the affected limb and measuring change, there are a number of subjective clinical metrology instruments (CMIs) in general use. These take the form of owner completed questionnaires designed to measure the functional limitation imposed by the disease and include the Canine Brief Pain Inventory (CBPI), Liverpool Osteoarthritis in Dogs (LOAD), Helsinki Chronic Pain Index (HCPI) and the Canine Orthopedic Index (COI) (41).

However, in 2006 the Canine Outcome Measures Program (COMP), formed with the intention of providing mechanisms and tools for improving the quality and impact of clinical studies in veterinary orthopedics, published guidelines which suggested that, in addition to at least one functional outcome, such as kinetics, kinematics, activity monitors, and/or an owner-reported CMI, all studies should include a validated health-related quality of life (HRQL) outcome measure (42).

Health-related quality of life instruments can be specific, focusing on particular conditions (disease specific), or they can be generic, designed for use in a variety of contexts. Disease specific instruments may be more sensitive to clinical change but generic instruments have been used successfully to quantify a range of impacts related to specific diseases including OA in people (43). Of the three currently available canine generic instruments (44–46), only VetMetrica™ (46) is validated for use in sick dogs. VetMetrica™ is an online behavior-based structured questionnaire instrument, designed to be completed on a computer or any mobile platform by the dog owner in around 5 min (46). It generates a HRQL profile for the dog with scores in four domains of QOL and has been used previously to measure the improvement in a group of dogs with OA treated with NSAIDs (47), and with RM (48).

While the majority of studies report a statistically significant change following treatment, responsiveness in a clinical measurement instrument is that property which ensures that the instrument can detect differences in health status that are important to the clinician and/or to the patient/dog owner and these need not be statistically significant (41). Responsiveness can be quantified by calculating a minimum important difference (MID) which is defined as “the smallest difference in score in the outcome of interest that informed patients or informed proxies perceive as important, either beneficial or harmful, and which would lead the patient or clinician to consider a change in the management” (49). The MID has been published for each domain of QOL in the VetMetrica™ instrument (50). In those measurement instruments where there is no MID published, clinical significance can be demonstrated by calculation of an effect size between control and treatment groups or between pre- and post-treatment groups (51).

Despite the extensive research and considerable promise shown by RM for treatment of MSD in canine and equine patients (19, 52), the origins, processing, and quality of stromal cells and PRP differ amongst the various treatment protocols, making it difficult to compare studies, a problem highlighted by Guest et al. (53). Additionally, there are no reports of large studies in which a variety of dog breeds with naturally occurring MSD were treated with RM. Data regarding protocols, duration of treatment responses and use in natural disease states such as OA in companion animals are also lacking (19). The present study addresses many of these concerns through its use of several validated outcome measures in a large sample of client-owned dogs with non-responsive chronic MSD recorded up to 4 years. The aim of this study was to investigate the efficacy of autologous MSC treatment for previously unresponsive chronic degenerative MSD in dogs, by retrospectively analyzing data from the clinical records of a single veterinary practice specializing in RM.^1^ As such, this is an example of real-world data (RWD) which is defined by The Association of the British Pharmaceutical Industry as “data that are collected outside the controlled constraints of conventional randomized clinical trials (RCT) to evaluate what is happening in normal clinical practice” (54). While RCTs provide evidence of efficacy, studies using RWD have greater generalizability and give evidence of effectiveness in real-world settings (55). Following on from the US Food and Drug Administration (FDA) Real-world Evidence (RWE) program, RWD has become increasingly important in human healthcare to support a wide range of healthcare and regulatory decisions (56, 57). In addition to data generated by a selection of objective outcome measures, owner-reported HRQL data were obtained. Our hypothesis was that treatment with RM would produce both significant statistical and clinically relevant improvement in MSK function and QOL in affected dogs.

## 2. Materials and methods

### 2.1. Ethical statement

This study was performed in line with the Mars scientific research and engagement policy (https://www.mars.com/about/policies-and-practices/scientific-engagement). All dog owners gave written informed consent for anonymised pet data to be used in the study. The RCVS Ethics Review Panel approved the study.

### 2.2. Case details and inclusion criteria

Medical records of client-owned dogs diagnosed with chronic MSD that were treated with RM from September 2017 to May 2021 were reviewed. Inclusion criteria for data collection were as follows: Dogs with MSD for which conventional therapy had been unsuccessful according to the referring veterinary surgeon; and whose owners had completed at least two sets of HRQL assessments; and at least one objective outcome measure. All cases were managed by a single clinician (author AA) who had extensive experience of RM having treated more than 600 dogs over 9 years with autologous MSCs, ensuring consistent clinical assessment, treatment and objective measurement outcomes. For each dog, all HRQL assessments were completed by the same owner.

On initial presentation all dogs underwent a full orthopedic and neurological physical examination including stance analysis and gait observation. Radiography and musculoskeletal ultrasound (MSK US), which was performed in paired joints allowing comparisons of measurements and echogenicity of anatomical structures between contralateral limbs, were undertaken to reach a definitive diagnosis. Confirmation that conventional therapy for the patients MSD had been unsuccessful was determined by reviewing the clinical history and demonstrating persistent pain, lameness or disability despite appropriate analgesic medications and previous interventions.

Osteoarthritis severity was graded on the basis of radiographic findings with additional clinical and ultrasound descriptions, using set criteria defined by author AA (Supplementary material 1). Where there was no radiographic evidence of OA, but ultrasound revealed a joint effusion and synovitis, and or cartilage defects, then these joints were classified as having Grade 1 OA.

Clinical and diagnostic findings were discussed with the owners and various treatment options including surgery, physical therapies, and altered pharmacological management, explained. Where a treatment plan was decided on that involved RM, the dog underwent fat harvest for stem cell extraction and culture. Patients whose treatment plan did not involve RM were returned to the referring veterinary surgeon for management.

For patients already receiving physical therapies (such as physiotherapy or hydrotherapy) this was discontinued after fat harvest and not resumed until after the post-treatment examinations at 12 weeks for single treatments and 18 weeks for two treatments. Physical therapies were not started until sufficient healing had been achieved in tendons and ligaments (as assessed by MSK US) and pain was determined to be under control.

## 2.3. MSC collection and preparation

### 2.3.1. Adipose tissue and blood collection

Dogs were anesthetized using a standard protocol and, following surgical preparation of the site, at least 5 g of adipose tissue was harvested from the falciform ligament *via* a cranial laparotomy incision cranial to the umbilicus. The adipose tissue was placed immediately into a sterile container with saline and sealed. An uncoagulated blood sample, the volume of which was determined by the quantity of stem cells required for culture and the patient's body weight and blood volume, was collected under aseptic conditions from the jugular vein. The adipose tissue and blood were packaged into cool boxes with chilled packs (4–10°C), sent to a specialized veterinary cell culture laboratory^2^ and processed within 24 h of harvest.

### 2.3.2. Tissue processing and MSC culture

At the laboratory, MSCs were extracted from the adipose tissue samples as described by Smitzi et al. (58). Once in culture at passage 0 they were incubated at 37°C in a humidified incubator with 5% CO_2_ in Expansion Medium (EM) (high glucose Dulbecco's Modified Eagles Medium (DMEM, ThermoFisher Scientific, Swindon, UK) with 10% Fetal Calf Serum (FCS, TCS Biosciences, Buckingham, UK) and 1% antibiotic and antimycotic (ABAM, ThermoFisher Scientific), which was changed every 2–3 days. The cultures were passaged until sufficient MSCs were present for treatment but were not passaged beyond passage 4. Twenty-four hours before the MSCs were due to be harvested, the flasks were washed twice with Phosphate Buffered Saline (DPBS, ThermoFisher Scientific) and incubated in a pre-treatment medium for 24 h as described above. For proprietary reasons, the constituents of the pre-treatment medium are not described.

Treatment vials were prepared by washing the attached cells with DPBS and then incubating for 5–10 min at 37°C in TrypLE^TM^ (ThermoFisher Scientific) until the cells were in suspension. The suspended cells were washed in DPBS by centrifugation at 1500 g. The final cell pellet was resuspended in Autologous serum with 10% saline and 10% Dimethyl Sulphoxide (DMSO, Sigma-Aldrich, Gillingham, UK) at a cell concentration of more than 2.5 million cells per ml for intra-articular treatment and 10 million cells per ml for epidural treatments. Intravenous doses were created at a concentration requested by the treating veterinary surgeon. Two milliliters of cell suspension were placed into a CellSeal^TM^ vial (Sexton Biotechnologies, Indianapolis, USA), sealed using a tube sealer, labeled and cryopreserved using a CoolCell^TM^ (Azenta Life Sciences, Manchester, UK) container in a −80°C freezer. Two 50 μl samples of the final MSC suspension were reserved for quality control. The cryopreserved treatment vials were maintained at −80°C during transportation and storage (for <4 weeks) at the clinic until they were required for injection. Internal quality control has confirmed that the cells remain viable for at least one month when stored at −80°C.

### 2.3.3. MSC characterization and quality control

Trilineage differentiation was demonstrated based on the methods described by Russell et al. (59). For chondrogenic differentiation MSCs at passage 3 were seeded at 2 x 10^5^ cells in 0.1 ml low glucose EM per well on a low adhesion Nunc u-bottomed 96 well plate (ThermoFisher Scientific) and left to settle in the incubator as described above. After 48 h the EM was removed, spheroids washed twice with PBS and then 0.1 ml StemPro Chondrogenesis Differentiation medium (ThermoFisher Scientific) was added per well. Control wells were maintained in low glucose EM throughout. Plates were maintained in the incubator for 21 days with medium changed every 2–3 days. Spheroids were stained with Alcian Blue (ThermoFisher Scientific) following fixation with paraformaldehyde, then washed with 0.1 N HCl (Sigma-Aldrich), neutralized with distilled water and visualized under light microscopy. The spheroids were then mounted on glass slides, compressed under a glass coverslip and imaged.

For osteogenic differentiation MSCs at passage 3 were seeded at 2 x 10^5^ cells per well in 1 ml low glucose EM on a 24 well plate (Sarstedt, Leicester, UK) and incubated as described above with the medium changed every 2–3 days until they reached 80% confluence. The medium was removed, the cells washed with DPBS and then test wells were treated with 1 ml StemPro™ Osteogenesis Differentiation medium (ThermoFisher Scientific) and control wells with low glucose EM for 14 days with medium changed every 2–3 days. The monolayer of cells was fixed with 10% Formalin (Sigma-Aldrich), washed with demineralised water and then 1 ml 40 nM Alizarin Red S (Sigma-Aldrich, UK) was added to each well and incubated at room temperature in the dark for 30 min. The stain was washed off with deionised water before visualization with an inverted microscope.

For adipogenic differentiation MSCs at passage 3 were seeded at 5 x 10^4^/well onto a tissue culture coated 96 well plate (Sarstedt) in 0.1 ml StemMACS AdipoDiff Media (Miltenyi Biotec, Bisley, UK), control wells were seeded at the same density in low glucose EM. The plate was incubated as described above and the medium changed every 2–3 days. After 14 days the cells were fixed with 4% paraformaldehyde, washed with DPBS and stained with Oil Red (Sigma-Aldrich) washed twice with deionized water and visualized with an inverted microscope.

Immunophenotyping was carried out based on the methods described by Krešić et al. (60) and Ivanovska et al. (61). Briefly, seven cell lines of MSCs (taken at random from the bank of MSCs stored at the lab) were cultured to passage 2 and when 80% confluent were trypsinised as described above. The cell pellet was resuspended in 1 ml of 4% paraformaldehyde (Sigma-Aldrich) for 15 min and washed with DPBS. For each antibody 1–2 x 10^5^ MSCs for each cell line (*n* = 7) were transferred to conical Eppendorf tubes. The fixed MSCs were then resuspended in 1:100 diluted canine antibodies in 2% Bovine Serum Albumin (BSA, Sigma-Aldrich)/DPBS, incubated in the dark for 30 min at room temperature, washed twice with DPBS, resuspended in ice cold DPBS and read on the BD C6 flow cytometer (BD Biosciences, Wokingham, UK) with a 1 x 10^4^ event limit (see Table 1 for the antibodies used for immunophenotyping). These data were analyzed using BD Accuri C6 Plus software (version 1.0.23.1; BD Biosciences). The samples were run with unstained controls and the corresponding isotope controls.

**Table 1:** Antibodies for immunophenotyping.

**Primary antibodies**	**Isotope controls**
CD44 Monoclonal Antibody (YKIX337.8), FITC	Rat IgG2a kappa Isotype Control (eBR2a), FITC
CD90 (Thy-1) Monoclonal Antibody (YKIX337.217), PE	Rat IgG2b kappa Isotype Control (eB149/10H5), PE
CD34 Monoclonal Antibody (1H6), PE	Mouse IgG1 kappa Isotype Control (P3.6.2.8.1), PE
CD45 Monoclonal Antibody (YKIX716.13), FITC	Rat IgG2b kappa Isotype Control (eB149/10H5), FITC
MHC Class II Monoclonal Antibody (YKIX334.2), APC	Rat IgG2a kappa Isotype Control (eBR2a), APC

Primary conjugated canine antibodies and their corresponding isotope controls. All antibodies were eBioscience™ from Thermofisher.

Release criteria for the MSC treatment vials required all batches (i.e., every individual MSC culture) to pass the final culture morphology check, a 4-day microbiology test and a post-cryo viability and cell count. The results of these tests were supplied to the treating veterinarian (AA) on a certificate of analysis. Quality control tests are detailed in Supplementary material 2.

## 2.4. PRP processing

Depending on body weight and amount of PRP required, 25 or 50 ml of anticoagulated blood was collected and total white blood cell (WBC), differential counts, red blood cell (RBC) concentration and platelet concentration determined using an inhouse Idexx Procyte hematology analyser (Idexx Laboratories, Westbrook, ME, USA), calibrated according to the manufacturer's recommendations. The anticoagulated blood was processed, according to the manufacturer's instructions, using a canine validated system (PurePRP Kit, Companion Regenerative Therapies, Newark, DE, USA). One half to 1 ml of PRP was dispensed into 1 ml syringes for injection and a sample of the PRP was analyzed using an inhouse Idexx Procyte hematology analyser (Idexx Laboratories, Westbrook, ME, USA) as before. The PRP product composition was confirmed for each sample. The increased platelet cell count from whole blood was calculated by dividing the PRP platelet count by the whole blood platelet count to give a concentration factor specific to each patient.

## 2.5. Treatment protocol

At the clinic, MSC treatment vials were allowed to defrost at room temperature. The exact number of cells required for treatment was determined from the certificate of analysis which detailed the cell count in millions per milliliter, % viability post thaw, and release criteria passed.

Dogs were sedated with a combination of medetomidine (Sedator, Dechra Veterinary Products, Shrewsbury, UK) and methadone (Comfortan, Dechra Veterinary Products, Shrewsbury, UK) administered intravenously, or given a general anesthetic, as before, for treatment with a combination of stem cells and PRP, or MSCs alone in the case of LS epidural injection.

Treatment sites were aseptically prepared and joint injections performed using standard approaches. For intra-articular treatment, fluid was aspirated to ensure correct needle placement and to remove excessive fluid prior to injection of cells, and MSK US was used to guide treatments to target specific pathology in muscle, tendon or ligaments. LS epidural injections were performed with a spinal needle using a standard approach and, in those cases where spondylosis was present in the cervical and thoracic regions, IV stem cells were infused in addition to the epidural injection at the LS junction.

For each joint or tendon lesion, > 2.5 million stem cells were injected at each location. In the case of LSD > 10 million cells were injected in the epidural space at the LS junction and where IV treatments were administered, 1 million cells per kg bodyweight were given *via* slow IV infusion. MSCs and PRP were combined and mixed in a sterile manner immediately prior to injection for intra-articular and tendon treatments. MSCs alone were injected into the epidural space or when intravenous treatments were required. Adverse reaction to any treatment was recorded in the clinical record.

Treatment sites received Class IV laser therapy (Companion Therapy Laser CTC-15, LiteCure, LLC, DE. USA) directly after injection and a further five sessions were completed over the following 3–6 weeks. Laser therapy (LT) was applied using a laser-contact ball by continuously moving the head in a grid pattern over the entire treatment area as per the manufacturer's recommendations. Laser power and duration of treatment was tailored to each individual depending on body weight, body condition, hair length, hair color and skin color. The dose provided to each treatment area was 10 joules/cm^2^.

All dogs were re-assessed clinically at approximately 6, 12, and 18 weeks following initial treatment and then every 3–6 months. At 12 weeks, in the case of severe pathology (i.e., Grade 3/5 OA or greater) or where dogs had received surgical arthroscopic intervention of the elbow prior to referral to author (AA), a second treatment of MSCs alone was administered. Similarly, at 12 weeks a repeat treatment with MSCs was administered where MSK US indicated that healing of treated soft tissue structures was incomplete. A single laser therapy treatment was provided at the time of injection to the treatment area when a second treatment was performed. In those dogs that received additional treatment more than 12 months after their previous treatment, the initial treatment protocol was followed. The treatment protocol has been summarized in Table 2.

**Table 2:** A summary of the treatment protocol used in all cases depending on the diagnosed pathology and severity in individual cases.

**MSD diagnosed**	**Initial treatment with MSCs (>2.5 million cells) and PRP**	**Initial treatment with MSC alone**	**Follow-up MSC (>2.5 million cells) treatment 3 months after initial treatment**	**Epidural administration of MCSs (10 million MSCs)**	**IV administration of MSCs (1 million cells per kg bodyweight)**	**Laser therapy (10J/cm^2^) at treatment site performed at time of each injection**	**3–6 week laser therapy course of five sessions on all treatment sites following initial treatment only**
OA grade 1–2/5 with or without dysplasia	✓					✓	✓
OA grade 3–5/5 with or without dysplasia	✓		✓			✓	✓
ED with previous arthroscopic interventions and OA grade 1–5/5	✓		✓			✓	✓
Soft tissue pathology (including tendons and ligaments)	✓					✓	✓
Soft tissue with incomplete healing identified by MSK ultrasound at 3 months post initial treatment			✓			✓	
LSD		✓		✓		✓	✓
Spondylosis affecting the cervical, thoracic and lumbar spine				✓	✓	✓	✓

### 2.6. Diagnostic imaging

Musculoskeletal US formed part of all clinical assessments and was used to evaluate muscles, tendons, ligaments, caudal lumbar intervertebral discs (IVD) and intraarticular structures including the joint capsule, volume of joint fluid, and changes to the synovium and articular surface integrity. Where pathological changes were unilateral, comparison was made with contralateral structures, using standard views. Tendons and their entheses were evaluated for enlargement by measuring their cross-sectional areas in specific anatomical locations and comparing to the contralateral structure. Elastography was used to image the elastic properties of tendons to confirm presence of fibrosis or scar tissue. Caudal lumbar IVD were evaluated by comparison with adjacent discs in order to measure thickness of the annulus fibrosus and echogenicity of the nucleus pulposus. Where there was extensive mineralization of soft tissue structures, in addition to MSK US, a repeat radiograph was taken of the affected area 12 weeks following initial treatment to determine the extent of any remaining abnormal mineralization. Where clinical assessment indicated the requirement for further treatment with RM, radiographs were taken and MSK US performed if appropriate, to establish if further or alternative pathology had developed.

### 2.7. Clinical outcome measurements

Objective outcome measures comprised part of the pre-treatment assessment and were repeated at all subsequent clinical assessments. Not all outcome measures were recorded for each patient where the clinical examination or diagnosis did not warrant it. For example, patients not suffering from LSD did not have PA performed and patients only suffering from LSD did not have joint goniometry performed.

#### 2.7.1. Stance analysis

A weight distribution platform (Companion stance analyser, LiteCure LLC, Newark, Delaware, USA) was used according to the manufacturer's instructions to measure percentage of weight distribution through each of the four limbs. Normal (target) weight distribution was taken to be 30% for each thoracic limb and 20% for each pelvic limb (29). Since many dogs in the study had bilateral MSD, an overall deviation from normal weight bearing was calculated by summing the absolute deviations of recorded from target values for each limb. Thus, zero would represent normal weight bearing, and larger values increasing deviation from normal. This value not only gives a value of offloading but also compensatory overloading and gives a better measure where multiple limb MSDs are present.

#### 2.7.2. Goniometry

The dog was positioned in lateral recumbency, and an appropriately sized universal plastic goniometer was used to measure full flexion and extension of the affected joint. The pivot point of the goniometer was placed over the center of motion of the joint and its arms aligned along the bone axes proximal and distal to the joint being measured. The proximal arm of the goniometer was held *in situ* whilst the joint was fully flexed and extended. The values were read in degrees from the goniometer and recorded in the clinical record. The ROM was calculated by subtracting the flexion angle from the extension angle. Further information regarding anatomical landmarks for goniometer placement are detailed in Supplementary material 3 and images of correct placement have been published previously (30).

#### 2.7.3. Pressure algometry

When lumbosacral pain was detected during the physical examination by direct palpation, Lumbosacral flexion and on tail hyperextension, a pressure algometer (Force Ten FDX compact digital force gauge. Wagner instruments, Greenwich, CT, USA) was used to quantify the pressure pain threshold. The LS junction was identified by palpating the dorsal spinous processes of L7 and S1 vertebrae. Pressure was applied at a steady rate to the dorsal lumbosacral (LS) junction (L7-S1) at right angles to the skin and the peak force (PSI) applied in order to elicit a pain response (any one of the following—dropping away from the instrument, turning of the head, vocalization or lip licking) was recorded as an average of three measurements and rounded to the nearest whole number.

#### 2.7.4. HRQL measurement (VetMetrica™)

The same owner for each dog included in this study was requested to complete VetMetrica™ assessments prior to treatment, then at 2, 6, 12, and 18 weeks post first treatment; then every 6 months thereafter. Except for the 2-week assessment, which did not coincide with a clinical examination, the owners completed their assessment before each clinical examination to minimize potential bias.

VetMetrica™ behavior-based structured questionnaire instrument contains 22 items (questions) for the owner. These items are simple descriptive terms, which are either positive (words associated with healthy conditions) or negative (words associated with unhealthy conditions). Each descriptor is associated with a 7-point (0–6) scale, which allows the owner to rate the extent to which the term depicts their dog. For example, for the term “playful”, 0 represents “not at all playful and 6 represents “couldn't be more playful”. Accordingly, in the case of a positive item like “playful” a score of 6 implies very good HRQL, but the same score implies very poor HRQL when the item is negative, for example “lethargic”. A coded algorithm automatically transforms the owner responses to all 22 items into a HRQL profile for the dog which consists of raw scores (0-6) in 4 domains of QOL – Energetic/Enthusiastic (E/E), Happy/Content (H/C), Active/Comfortable (A/C), Calm/Relaxed (C/R). Summary scores in physical wellbeing (PWB) and emotional wellbeing (EWB) can be calculated by averaging the E/E and A/C scores (PWB) and H/C and C/R scores (EWB) (Author JR Personal Communication). To aid interpretation, these raw domain scores are optimized by normalizing them to the age-related healthy dog population, such that a score of 50 on a 0–100 scale represents the score for the age–related average healthy dog. Additionally, 70% of healthy dogs will score above a threshold set at 44.8 on the 0–100 scale (50).

In order to determine the clinical significance of improvements in HRQL domain scores, the difference in median values was calculated between pre–treatment and each successive post-treatment time window. A change equivalent or greater than the MID of 7 was considered clinically significant.

#### 2.7.5. Vet clinical assessment

Timed to coincide with each owner HRQL assessment, other than the 2 weeks post initial treatment, a veterinary assessment (Supplementary material 4) was completed by author (AA) who was blinded to his previous scores by the fact that scores were entered directly into the VetMetrica™ database rather than the clinical record. The veterinary assessment comprised a list of common canine diseases which, when present, were graded as mild/moderate/severe/end stage. A freeform box was provided to accommodate any disease not specified in the list. Additional questions were as follows: “on a scale of 0–10, with 0 being no impact and 10 being the most impact, please assess how much the dog's health status is reducing its quality of life (QOL)”, and “on a scale of 0 to 10 with 0 being no pain and 10 being the pain could not be worse, please indicate what amount of pain you feel the dog is suffering”.

#### 2.7.6. Analgesic usage

Analgesic usage was compared at two time periods: pre-treatment up to 180 days (c.26 weeks) before the first treatment (but excluding the date of treatment) and 24–48 weeks post-treatment. Where multiple records existed, the latest in the pre-treatment period (closest to treatment date) and the earliest in the post-treatment period (closest to 6 months) was selected.

### 2.8. Data handling and analysis

#### 2.8.1. Retrospective data collection

Patient data included signalment, history, diagnosis, prior treatments (including treatments at initial presentation), analgesic therapy, physical examination findings, diagnostic imaging results and objective outcome measurements (weight distribution at stance, joint angle goniometry measurements and pressure algometry readings) were extracted from the case record. Dogs were excluded from the analysis of each individual outcome measure if there were insufficient data recorded in the medical record pre- or post-treatment. Normalized scores in four domains of QOL calculated from the VetMetrica™ owner-reported health related quality of life (HRQL) questionnaires, and vet pain and QOL impact scores, were extracted from the VetMetrica Database. Ideally HRQL assessments corresponded with clinic visits and vet assessments, except at 2 weeks when there was no clinic visit. However, individual owner circumstances often dictated that this was not possible. Consequently, a 14-day interval was considered a reasonable cutoff point to maximize the probability that the dog's health status had not changed between owner assessments and clinic visits, and so all assessment pairs that had >14 days between them were excluded from the HRQL analysis.

#### 2.8.2. Statistical analysis

To determine if there was an improvement in objective outcome measures and owner reported health related quality of life following treatment with RM, data were divided into pre-treatment phase, and then into time windows 0–6 weeks, 7–12 weeks, 13–18 weeks, 19–24 weeks, 25–48 weeks, 49–78 weeks (18 months), and 79–104 weeks (24 months) post first treatment. Data beyond 104 weeks were not considered further because of low numbers.

Data were analyzed as a linear mixed model with time window as a fixed effect and dog within time window as a random effect. This analysis takes into consideration that there may be no, or multiple values per dog per time window. Significant effects (*p* < 0.05) were subsequently investigated using Tukey multiple comparisons. Data for LS pain were restricted to those dogs with LS disease. Likewise, joint ROM measurements were restricted to dogs receiving one or more treatments in that joint. For the analysis, the ROM values for right and left joints were averaged to provide a total ROM value for the joint. More limited data were available for the range of motion for stifle, hock, and carpi than for the other joints, so their analysis was supplemented by a further analysis just comparing pre-treatment with post-treatment (to 104 weeks) windows. Results are presented as boxplots of raw data for each time window.

To determine the relationship between change in stance and change in VetMetrica domain scores as well as Vet pain and QOL impact scores, deviation from perfect stance was matched to HRQL domain scores, vet pain and QOL impact scores if the interval between them was ≤ 14 days. For each HRQL, pain and QOL impact variable a linear mixed model was fitted with dog as a random effect and change in stance as a covariate.

The reduction in analgesic medication at two defined time points (pre- and post- treatment) was tested using Wilcoxon signed rank tests.

Cohen's effect size (d) was calculated for ROM and PA measures.

## 3. Results

### 3.1. MSC differentiation and immunophenotyping

**Figure 1:**
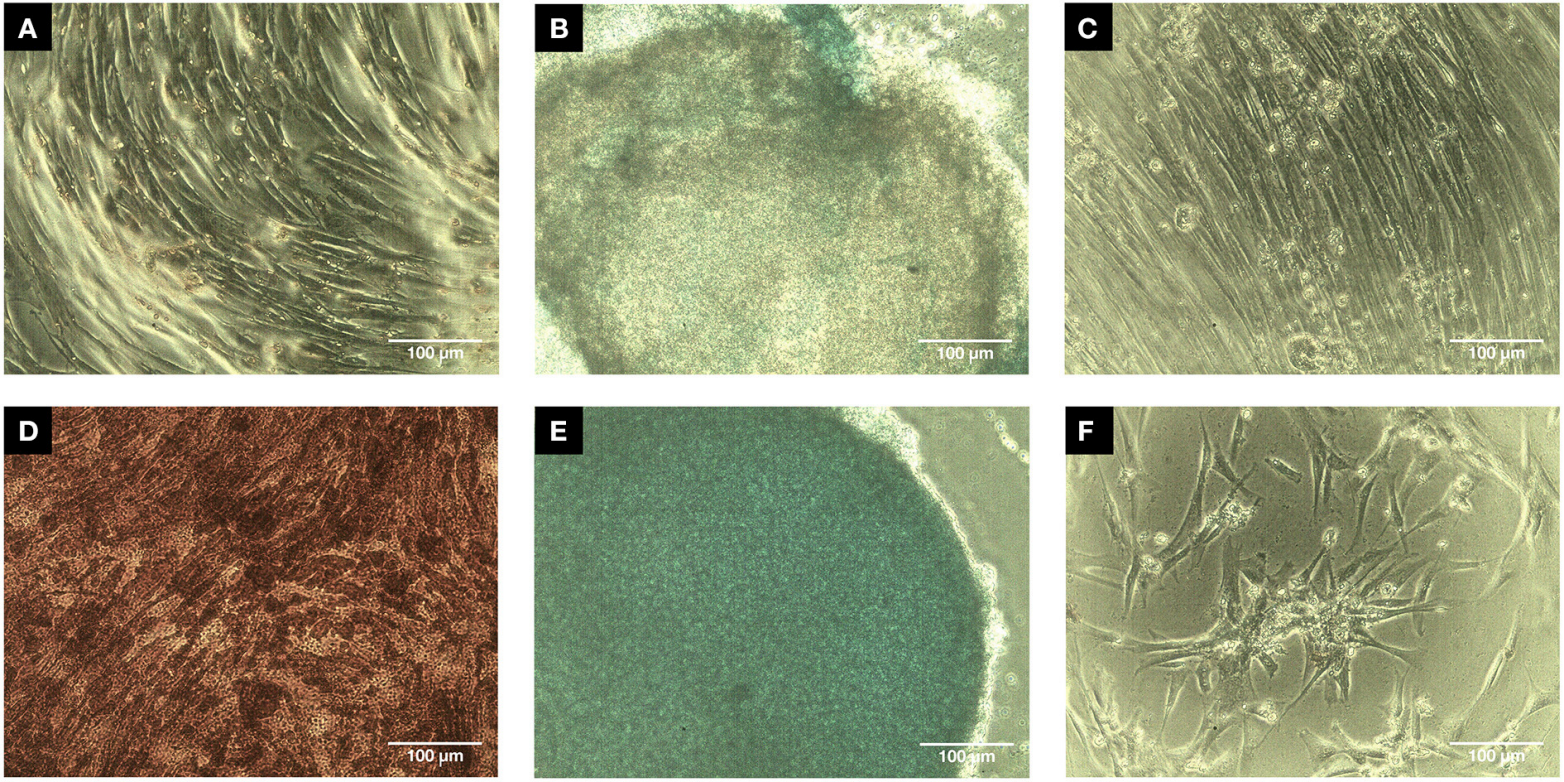
Trilineage assay micrographs (Scale bar = 100 μm). Passage 2 canine MSCs were successfully induced after 14 days into osteogenic differentiation **(D)** as mineralization was positively stained while the control **(A)** had no positive staining for Alizarin red. Similarly, chondrogenic differentiation was successfully induced after 21 days as indicated by intensity of positive Alcian blue staining **(E)** compared to the control **(B)** which only had partial positive staining. The adipogenic differentiation after 14 days was negative **(F)** with no Oil Red staining in comparison with the adipogenic differentiation control **(C)**.

**Figure 2:**
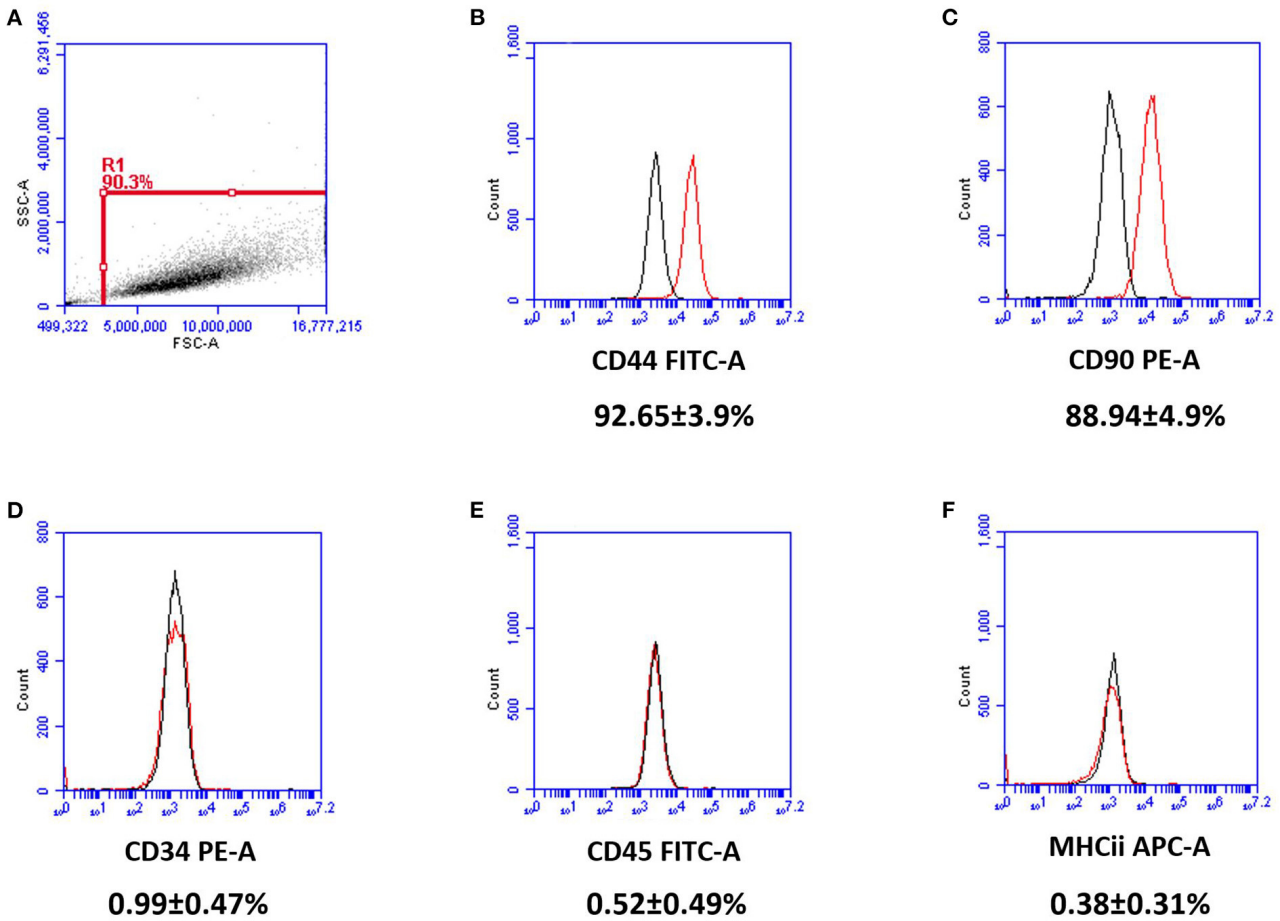
Flow cytometry results for passage 2 canine MSCs (*n* = 7). Cell surface markers were represented by the red histograms with isotope controls labeled black. Gating was set to R1 **(A)**. MSCs expressed CD44 **(B)** and CD90 **(C)**, while lacking expression of CD34 **(D)**, CD45 **(E)**, and MHCii **(F)**. Percentages denote the intensity of positivity of the CD markers with standard deviations.

Canine MSCs isolated and culture expanded for the dogs in this study conformed to International Society for Stem Cell Research standards (58) and the Position Statement for Veterinary MSCs (53) with respect to all criteria apart from Adipogenesis which did not occur under the conditions tested. Figure 1 shows that both the osteogenic and chondrogenic differentiation cultures stained positively compared with the corresponding controls but the adipogenic culture did not stain positively with Oil Red in comparison with the corresponding control. Figure 2 shows that the MSCs were positive for CD44 and CD90 whilst being negative for CD34, CD45 and MHCii.

### 3.2. PRP composition

On average the platelet concentration factor was found to be 6.3 times that of whole blood with a 98% reduction in RBCs and a 92% reduction in neutrophils when compared to whole blood from the same patient. This was consistent with findings of other investigators using the same system (62).

### 3.3. Study population

Data were collected from 245 dogs, median age 6.3, range 6 months−14 years, 136 males and 109 females of which 72 and 76 respectively were neutered (Supplementary material 5). A wide range of breeds was represented, and these are detailed in Table 3. Dogs were referred for treatment with RM due to the severity and generalized nature of their MSD that was unresponsive to traditional treatments. On presentation dogs were receiving multiple analgesic medications including adjuvant drugs as well as NSAIDs but were still exhibiting pain and lameness. Twenty-four dogs had previous surgery related to cranial cruciate ligament rupture, 24 had previous surgical arthroscopic intervention for elbow developmental disorders, and 2 had surgical treatment for osteochondrosis (OCD) of the shoulder.

**Table 3:** Range of breeds represented in 245 dogs treated with regenerative medicine.

**Breed**	***n***
Labrador retriever	79
Border collie	39
Mixed breeds	20
English cocker spaniel	14
German shepherd / Alsatian	13
English springer spaniel	12
Golden retriever	10
Jack/Parson russell terrier	6
Bearded collie, Border terrier, Newfoundland, Rottweiler, Gordon setter, Staffordshire bull terrier	3
Bichon frise, Boxer, Hungarian vizsla, Lurcher, Weimaraner, Standard poodle	2
Airedale terrier, Alaskan malamute, Australian cattle dog, Australian kelpie, Australian shepherd, Cavalier King Charles spaniel, Smooth coated collie, French mastiff/Dogue de Bordeaux, Golden retriever, Greyhound, Italian spinone, Lakeland terrier, Miniature poodle, Patterdale terrier, Pug, Flat coated retriever, Rhodesian ridgeback, Samoyed, Scottish terrier, Slovakian rough haired pointer, Spanish water dog, and Toy poodle	1

Two hundred and thirty-four dogs were diagnosed initially with OA. Grades of OA 1, 2, 3, and 4 were represented by 24, 99, 100, and 6 dogs respectively; a further five dogs were not graded on initial presentation. The remaining 11 dogs were suffering from tendinopathies and/or LSD in the absence of OA.

Dogs had between 1 and 8 treatments with RM. The frequency and percentage of dogs receiving treatment is shown in Table 4. Of the 106 dogs that received two treatments only, the second took place between 70 and 126 days (10–18 weeks) for 40 dogs and after 126 days for 66 dogs. The former group comprises patients receiving two treatments as part of the initial treatment protocol and for the latter group the mean (SD) of the days between treatments was 450.9 (321.4) with the second treatment being given due to clinical need. Supplementary material 6 indicates how many records/dogs were present in each time window for each analysis and includes tables of fitted means for each time window with letter codes from Tukey multiple comparisons such that means not sharing a common letter are significantly different (*p* < 0.05).

**Table 4:** The number of dogs receiving between 1 and 8 treatments and their percentage of the study population (*n* = 245).

**Number of treatments**	**Number of cases**	**% of study population**
1	53	22%
2	106	43%
3	50	20%
4	19	8%
5	11	5%
6	4	2%
8	2	1%

**Figure 3:**
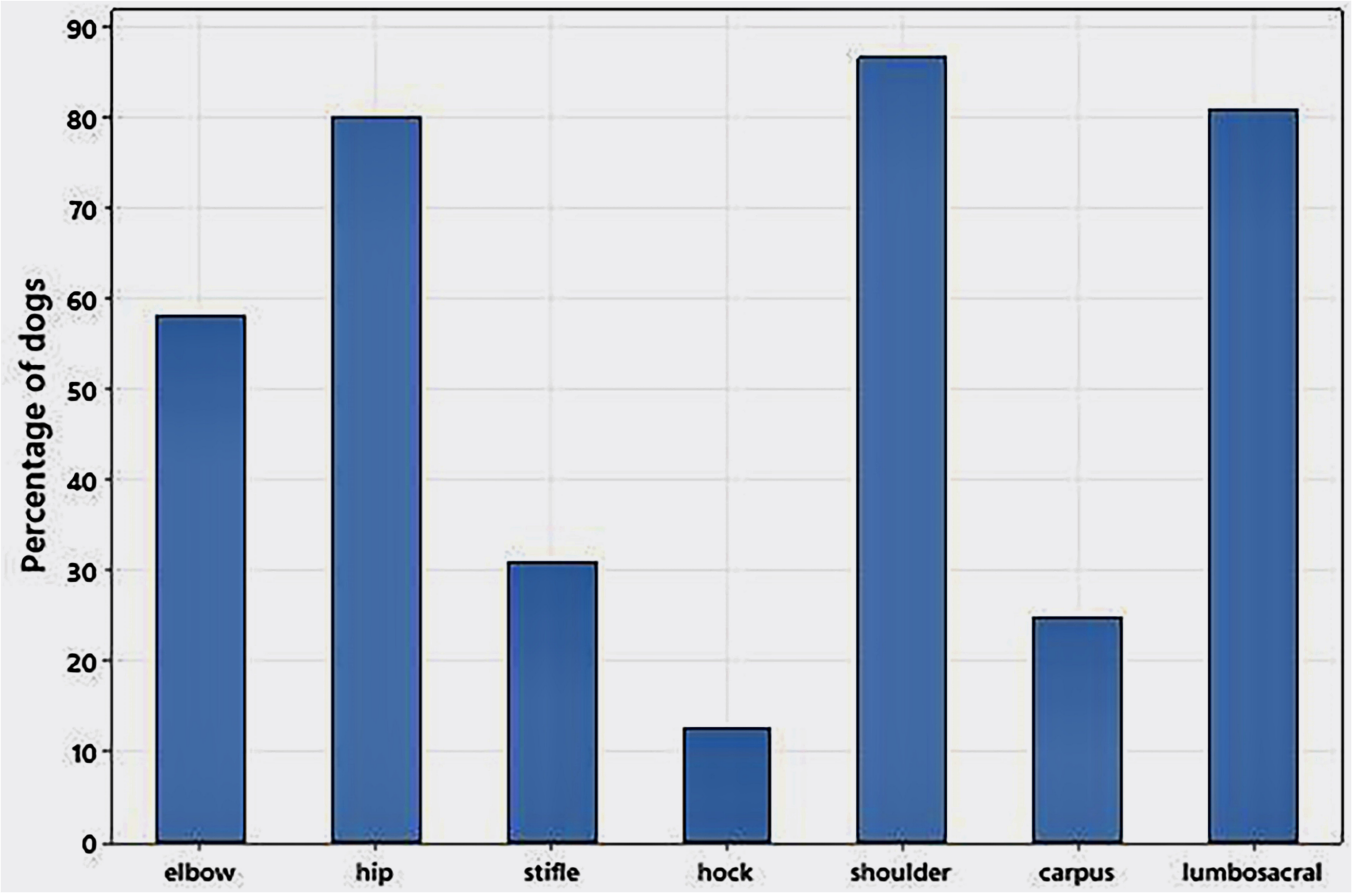
Proportion of 245 dogs with MSD treated with corresponding regenerative medicine to 104 weeks that received joint or lumbosacral treatments on at least one occasion. Multiple joints were treated in 89% of dogs and <1 % of dogs had only a single musculoskeletal disease treated.

Figure 3 indicates the proportion of dogs that received treatment in each of the six joints on at least one occasion, and the proportion of dogs treated at the lumbosacral region at least once.

The different MSDs treated in the study group are listed in Supplementary material 7.

### 3.4. Musculoskeletal ultrasound

**Figure 4:**
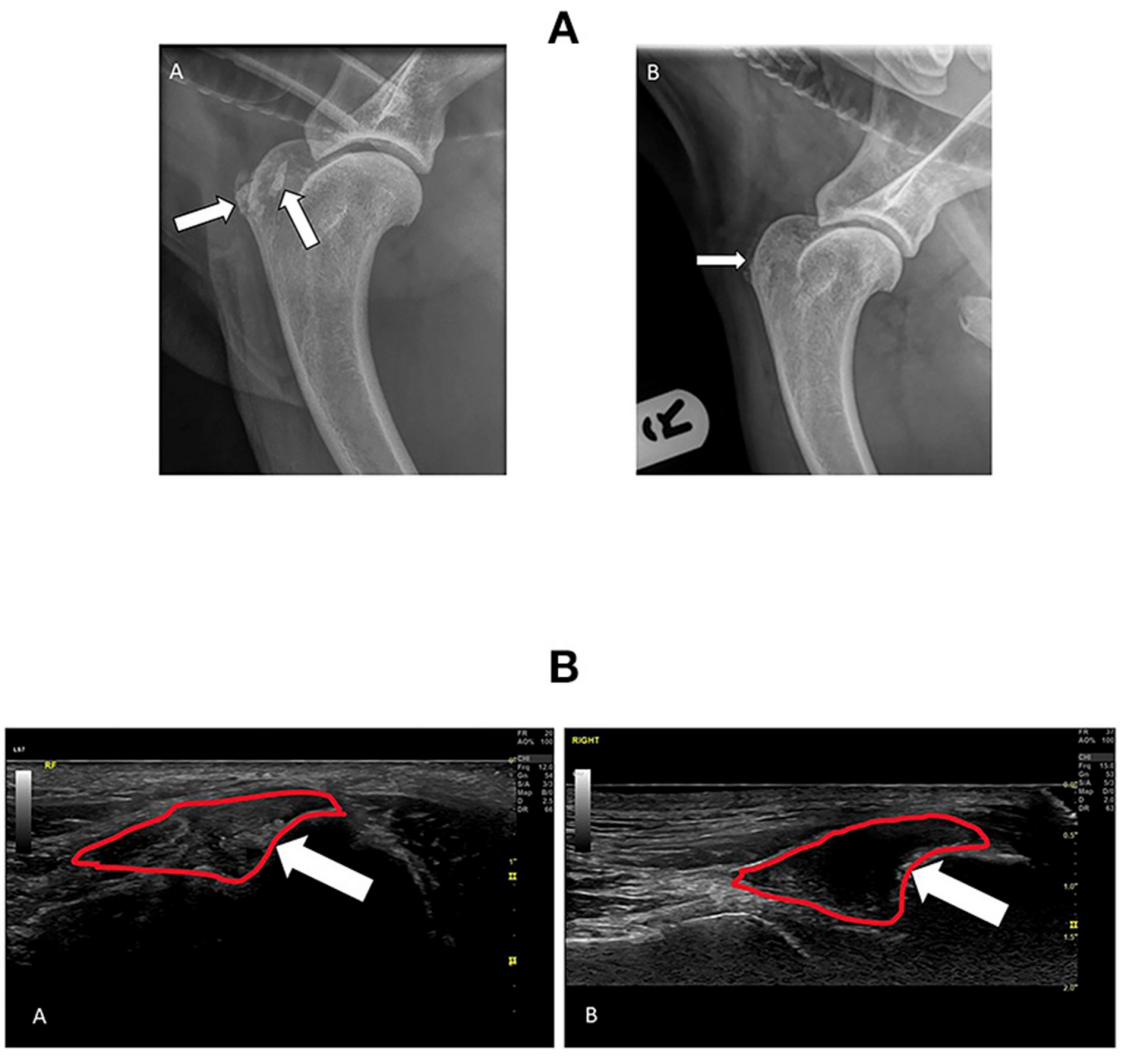
**(A)** Radiographs of the right shoulder from a 7-year-old male neutered Labrador with concurrent elbow dysplasia and OA showing extent of Supraspinatus (SS) tendon mineralization at the time of diagnosis (A) and 3 months following treatment with RM (B). The white arrows point to abnormal mineralization in the SS tendon and enthesis. Following treatment there has been a significant reduction in the extent of mineralization. **(B)** Ultrasound images of the right Supraspinatus (SS) tendon insertion before and after treatment in the same patient as in (A). The SS tendon enthesis is outlined in red and the white arrow points to areas of abnormal mineralization. (A), taken at the time of diagnosis, shows extensive mineralization of the enthesis and distal tendon with resultant acoustic shadowing. (B) was taken 3 months after treatment. This image shows a normal SS tendon and hypoechoic enthesis with only residual mineralization on the humeral attachment.

**Figure 5:**
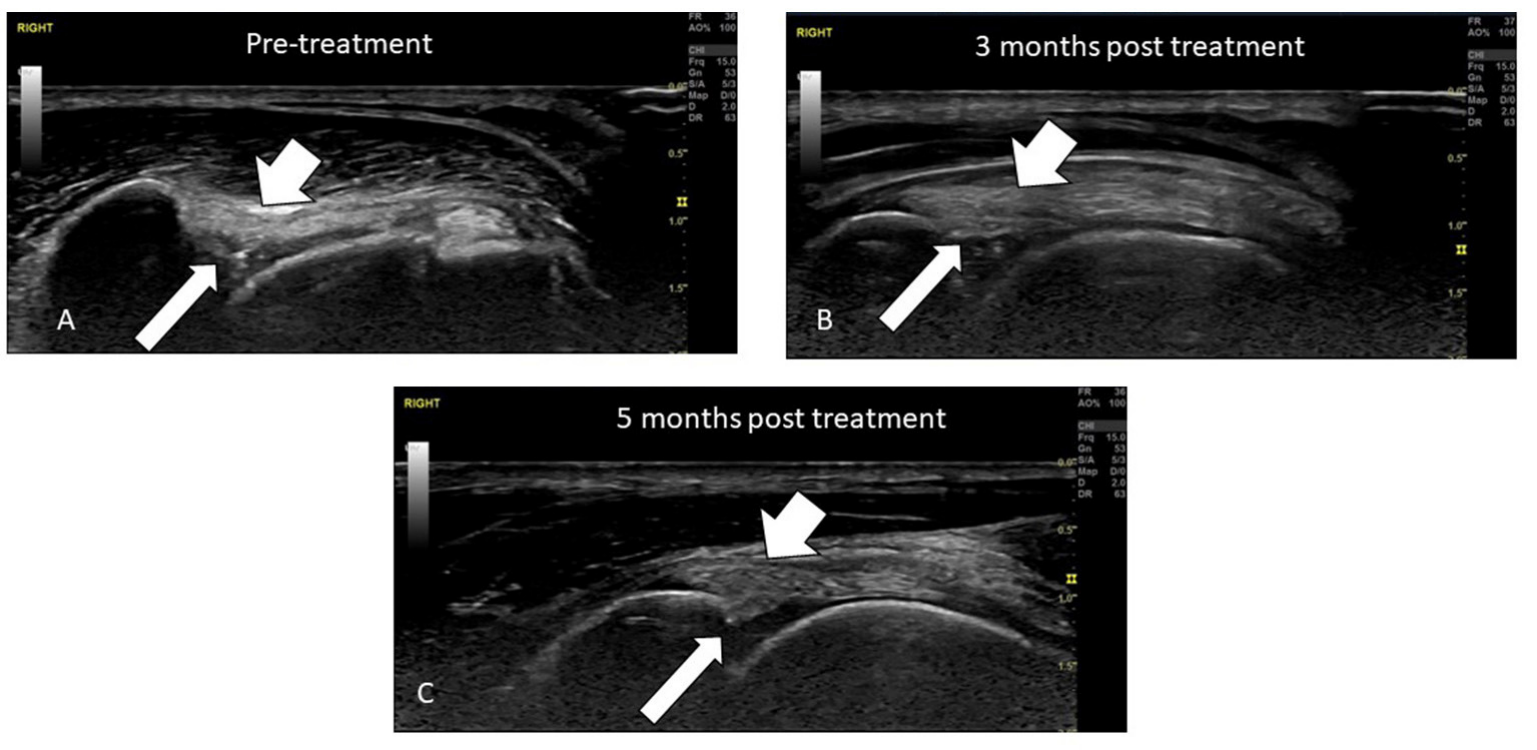
Ultrasound evaluation of a Biceps tendinopathy and partial Subscapularis tendon tear in 4-year-old agility dog before and after treatment with RM. The long arrows point to the position of the Subscapularis tendon and the short arrow the Biceps tendon. **(A)** Pre-treatment the Biceps tendon has extensive fibrosis and loss of a linear fiber pattern and enthesis and the Subscapularis tendon is partially torn with free floating fibers and fibrotic proliferation. **(B)** 3 months following treatment the Biceps tendon has shown extensive healing with a reduction in fibrosis and restoration of a more normal linear fiber pattern. The integrity of the Subscapularis tendon has improved but complete healing has not been achieved. A second treatment of MSCs was implanted. **(C)** 5 months following initial treatment and 2 months after the second treatment. The fiber patterns have improved with resolution of fibrotic infiltration, the biceps enthesis is normal and the integrity of the subscapularis has been restored.

In the main, repeat evaluations of treated tendons and other soft tissue structures performed with MSK US at 12 weeks post-treatment demonstrated considerable improvements in tendon fiber patterns and a reduction or elimination of inflammatory change, fibrosis and mineralization, as shown in Figure 4B. Before treatment, the SS tendon and its enthesis were severely degenerated with a loss of linear fiber pattern, fibrotic infiltration, and areas of extensive mineralization, but at 12 weeks post-treatment the tendon had resumed a normal fiber pattern and the enthesis was remodeled to a normal “sharks' fin” appearance with resolution of the fibrotic and mineralised portions and normal echogenicity. Figure 4A shows radiographic evidence of resolution of abnormal mineralization in the SS tendon insertion in the same patient at the same time points. In contrast, Figure 5 is an example of incomplete healing following treatment in a 4-year-old Border Collie with a sports-related shoulder tendon injury. The image on the left shows a chronic biceps tendinopathy and a partial tear of the subscapularis tendon with complete disruption of the fibers. At 12 weeks post-treatment, there was a considerable improvement in the fiber pattern, fibrosis and echogenicity of the biceps tendon and its enthesis. The integrity of the subscapularis tendon had improved but there was incomplete healing. Following a second treatment the subscapularis tendon had healed completely with a normal fiber pattern, without fibrosis/scar tissue and no free-floating tendon fibers were visible in the medial shoulder compartment.

### 3.5. Outcome measures

#### 3.5.1. Stance analysis

**Figure 6:**
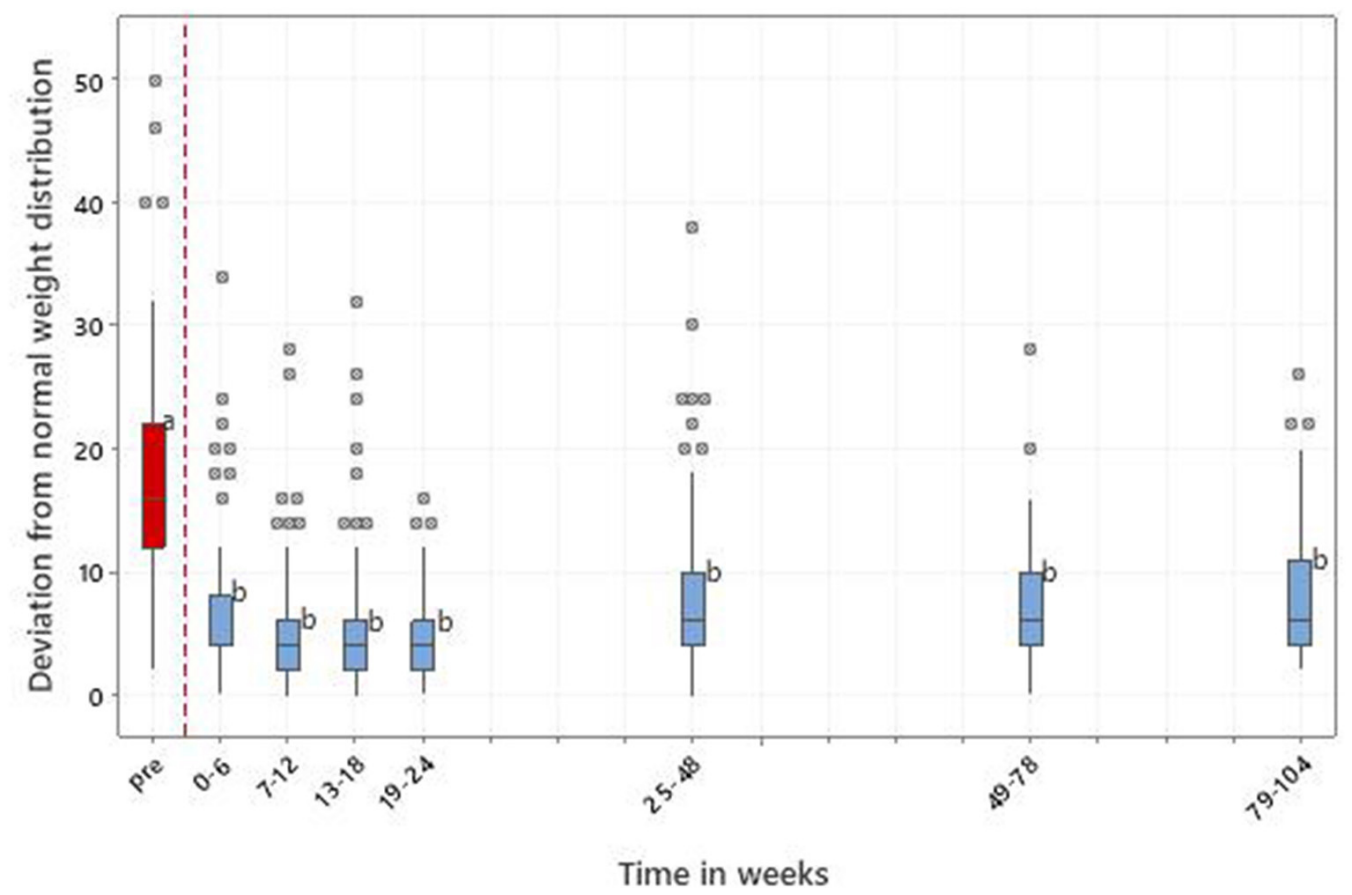
Results of stance analysis for 228 dogs with MSD treated with RM in time windows representing pretreatment (pre) and up to 104 weeks post initial treatment. 0 = perfect balance; > 0 = increasing imbalance. It should be noted that in this figure a single value of 78 in time window 7–12 weeks was removed in order to improve the use of the vertical space. This value related to a dog that had an acute traumatic incident resulting in non-weight bearing lameness, unrelated to its original diagnosed pathologies. The dog improved with rest and supplemental analgesia and its weight distribution normalized after 7 days. Letters adjacent to each bar indicate which time windows are significantly different (i.e., those not sharing a common letter).

There were 855 records from 228 dogs up to week 104. The results are shown in Figure 6. Results of the mixed model analysis showed that there were significant time window effects (*p* < 0.001); all post-treatment means were significantly lower compared to pre-treatment.

#### 3.5.2. Goniometry

**Figure 7:**
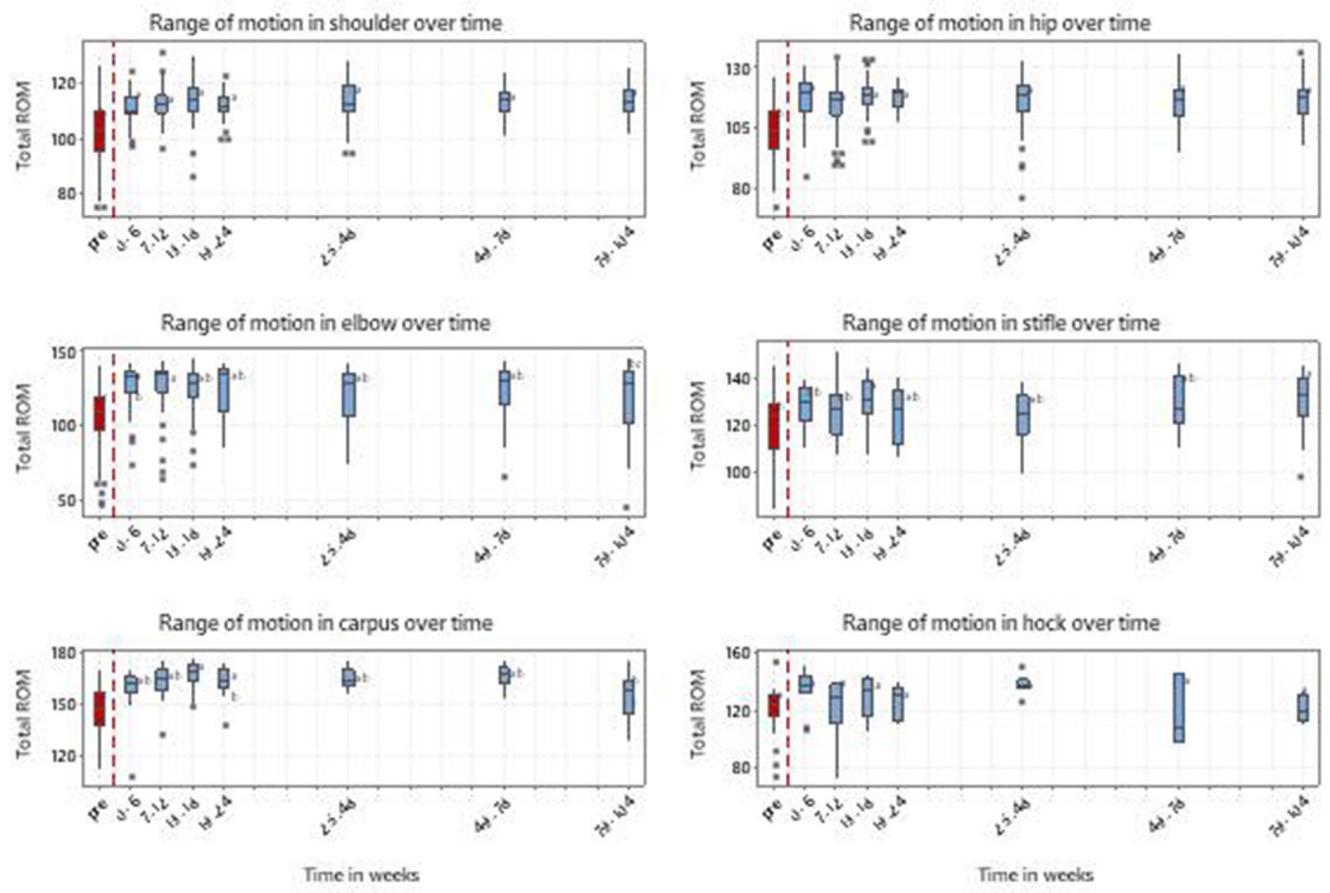
Range of motion in thoracic and pelvic limbs for 234 dogs with MSD treated with RM in time windows representing pretreatment (pre) and up to 104 weeks post initial treatment. Shoulder *n* = 191, Elbow *n* = 123, Carpi *n* = 51, Hip *n* = 158, Stifle *n* = 60 and Hock *n* = 28. Individual dogs may have received treatment to multiple joints. Letters adjacent to each bar indicate which time windows are significantly different (i.e., those not sharing a common letter).

There were 925 records from 234 dogs with a variable number of records/dogs for each measure, truncated at 104 weeks. Figure 7 represents the ROM in thoracic and pelvic limb joints, from pre-treatment to 104 weeks.

Analysis of individual joints indicated that there were significant time window effects (*p* < 0.001) with improvement at all time windows compared to the pre-treatment period for shoulder and hip; at all time windows except for 104 weeks for carpus and elbow (*p* < 0.001); and only at weeks 18 and 104 for stifle (*p* = 0.003). Time window effects for the hock did not reach significance (*p* = 0.062). For those less well-recorded joints where pre/post-treatment analysis was carried out, all were significant; carpus and stifle both *p* < 0.001, hock *p* = 0.020. The maximum improvement in ROM was 12, 14, 20, and 22 degrees for the shoulder and stifle, hip, carpus and elbow respectively.

In terms of effect size, changes in ROM between pre-treatment and the first 6 weeks post-treatment had a Cohen's effect size (d) ranging from 1.05 to 2.14.

#### 3.5.3. Pressure algometry

**Figure 8:**
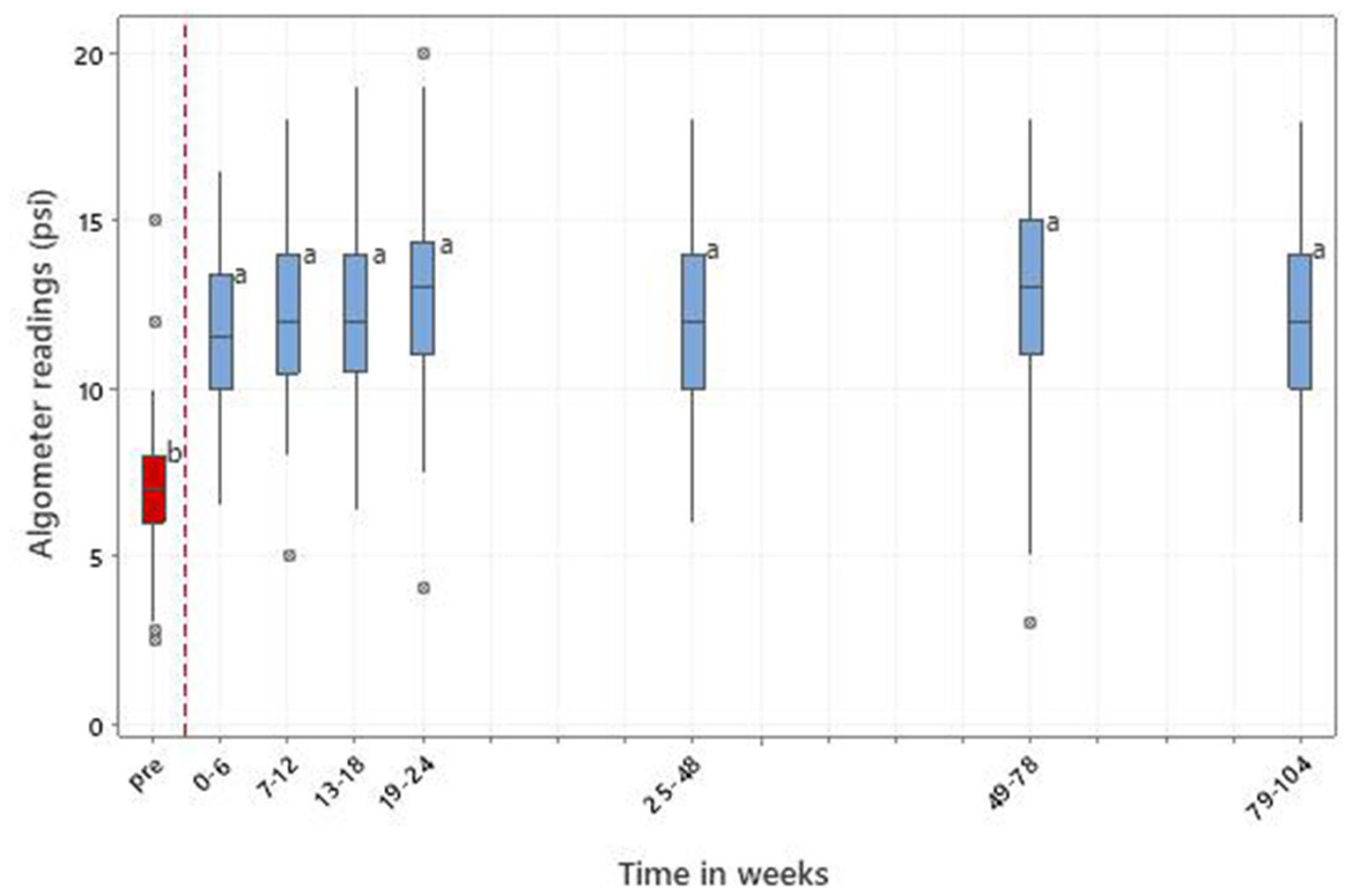
Pain threshold at the lumbosacral junction in 171 dogs with LSD treated with RM in time windows representing pretreatment (pre) and up to 104 weeks post initial treatment. Letters adjacent to each bar indicate which time windows are significantly different (i.e., those not sharing a common letter).

There were 568 records of LS pain from 171 dogs with LSD up to week 104. There were significant time window effects (*p* < 0.001) with all post-treatment means being significantly improved compared with pre-treatment (Figure 8). Cohen's effect size (d) was 2.20 for the same time period as ROM.

#### 3.5.4. HRQL measurement (VetMetrica™)

**Figure 9:**
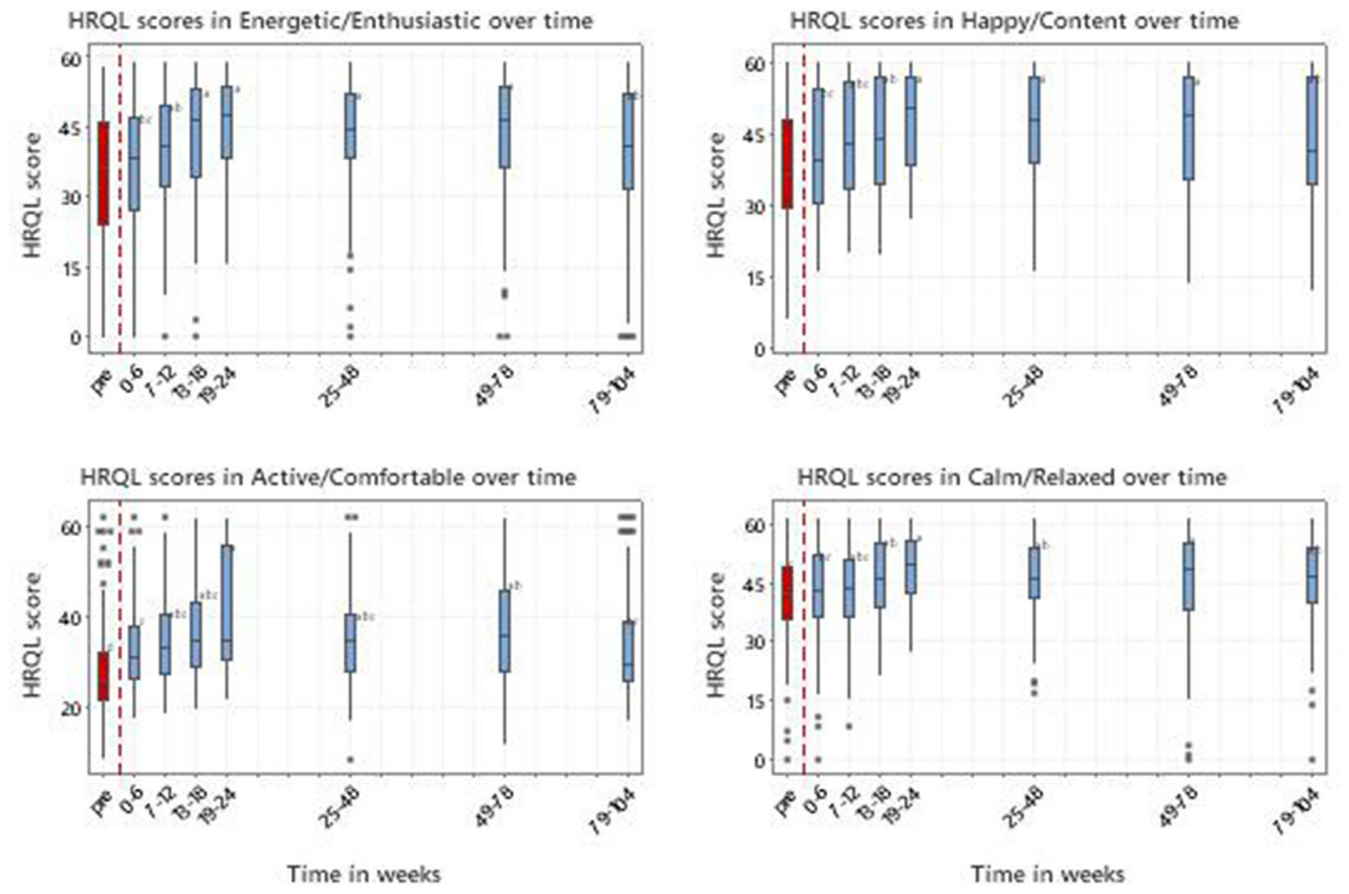
Scores in 4 domains of QOL (Energetic/Enthusiastic; Happy/Content; Active/Comfortable; Calm/Relaxed) for 212 dogs with MSD treated with RM in time windows representing pretreatment (pre) and up to 104 weeks post initial treatment. 50 represents the score for the average healthy dog and 70% of healthy dogs will score above 44.8. Letters adjacent to each bar indicate which time windows are significantly different (i.e., those not sharing a common letter).

A total of 954 owner Quality of Life (QoL) assessments in four domains were recorded from 212 dogs that had both QoL and treatment details up to week 104. Figure 9 shows the HRQL scores over time in all four domains. In E/E there were significant time window effects (*p* < 0.001) with all time window means after 6 weeks being significantly higher than pre-treatment. In H/C and C/R all post-treatment means were significantly higher than pre-treatment after 12 weeks (*p* < 0.001). In A/C all post-treatment means were significantly higher than pre-treatment (*p* < 0.001), with a slight reduction in later time windows.

From 13 to 78 weeks all improvements in median scores for E/E, H/C and A/C were considered clinically significant on the basis that they exceeded the MID of 7 (Supplementary material 8).

#### 3.5.5. Vet clinical assessment

**Figure 10:**
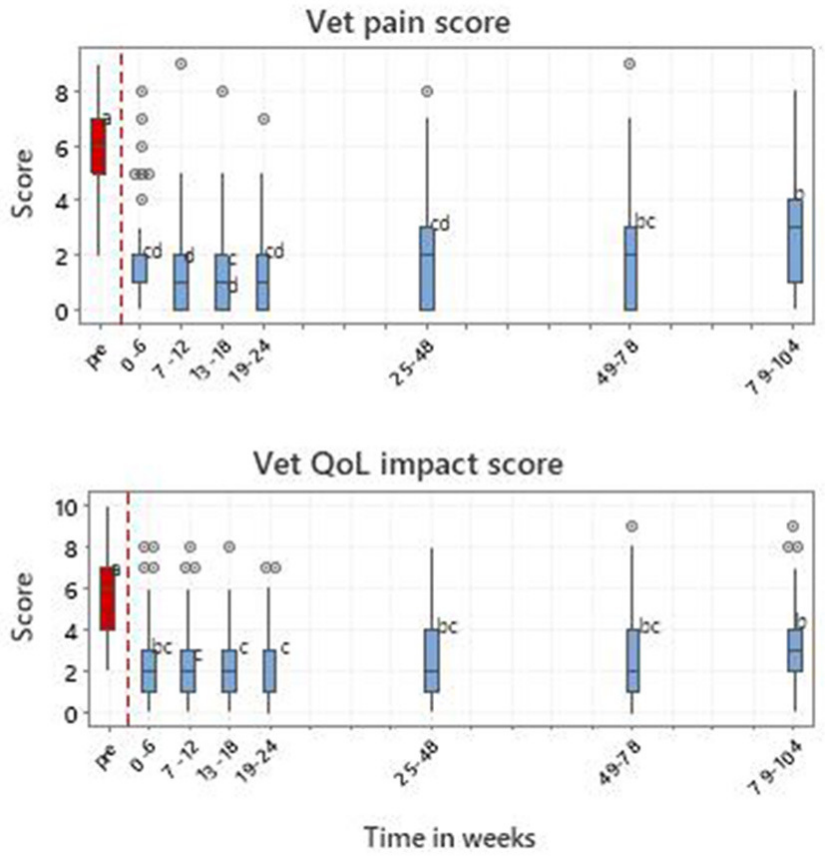
Vet-assessed pain and QOL impact scores for 223 dogs with MSD treated with RM in time windows representing pretreatment (pre) and up to 104 weeks post initial treatment. Letters adjacent to each bar indicate which time windows are significantly different (i.e., those not sharing a common letter).

Vet pain and QOL impact scores were recorded 906 times from 223 dogs up to week 104. Figure 10 depicts the scores over time for vet pain and QOL impact. There were significant time window effects (*p* < 0.001) with all post-treatment means being significantly improved from pre-treatment, but with a deterioration in later time windows.

#### 3.5.6. Relationships between change in stance and change in VetMetrica and vet assessment scores

**Figure 11:**
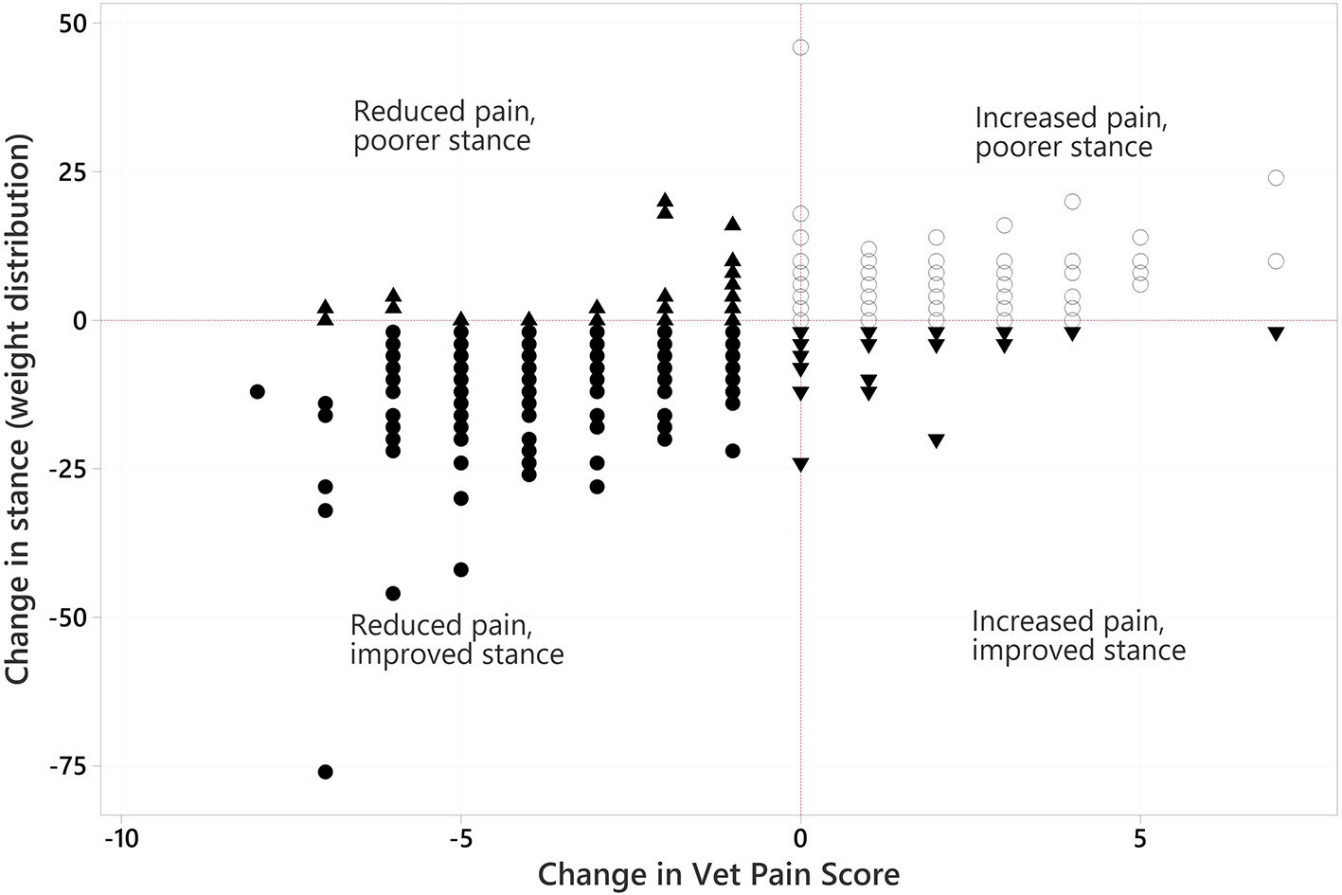
Scatter of changes in stance (weight distribution) plotted against changes in vet-assessed pain score. In each variable negative values reflect an overall improvement. Different symbols are used merely to indicate dogs in combinations of the improved/not improved categories of both variables.

There were change records from 123 dogs where the deviation from perfect stance was matched to HRQL domain scores within a 14-day period. These data showed that there was a significant relationship (*p* < 0.001) between the improvement in stance and the improvement in HRQL scores in all four domains. Similarly, the relationship between the improvement in stance and the decrease in vet-assessed pain (Figure 11) and QOL impact scores was significant (*p* < 0.001).

#### 3.5.7. Analgesic usage

A total of 118 dogs had analgesic records in both pre- and post-treatment periods. Mean dates of records were 41 days pre-treatment (range 1–159 days before), and 225 days post-treatment (range 170–336). The proportion of dogs receiving NSAIDs declined from 74 to 50%, the change was significant (*p* < 0.001) based on a Wilcoxon signed rank test. The proportion of dogs receiving adjuvants (such as Paracetamol, Gabapentin, Codeine, Amantadine and Tramadol) declined from 80 to 35%, and the mean number of adjuvants per dog declined from 1.58 to 0.64. Both were significant (*p* < 0.001) using Wilcoxon signed rank tests.

### 3.6. Adverse events

A small number of mild adverse reactions, including skin irritations following clipping and surgical preparation of the skin were recorded. Transient short lived (<36 h) joint flares following intra-articular injection, were also reported in <1% of the study population.

## 4. Discussion

This paper is the first to report the duration of improvement that can be achieved with multiple applications of quality controlled, culture expanded autologous MSCs to treat widespread MSD affecting joints, tendons, ligaments and pathology of the LS region, in a large sample (245) dogs that were exhibiting pain and lameness despite receiving multiple analgesic medications, including adjuvant drugs as well as NSAIDs. In contrast, Sanghani-Kerai et al. reported the results of 25 dogs that received intra-articular injections of MSC and PRP on a single occasion as part of their OA management, and only three of the 25 dogs had more than one joint treated, with outcomes recorded for only 24 weeks (63). Furthermore, this study is the first to utilize a standardized therapeutic protocol that included LT in addition to MSCs and PRP. Compared with the standard approach of using objective outcome measures to define the functional impact of treatment, this study was enhanced by the addition of a validated HRQL assessment and by the use of Cohen's d effect size to quantify clinical significance in addition to statistical significance.

The authors consider RM to be a targeted treatment approach that requires a holistic MSD diagnosis to treat all areas of pathology concurrently in order to achieve the best outcomes. In addition to a complete orthopedic and neurological examination, diagnostic imaging was used to reach a definitive diagnosis in all cases. Radiographs were used to assess bone and joint changes and MSK US was used to evaluate soft tissue structures. Radiographs of the shoulder are not diagnostic for shoulder tendinopathies unless extensive mineralization is present, and so MSK US allowed for greater detection of tendinopathy than if radiography had been used alone. Furthermore, because MSK US provides a means to evaluate joint capsule pathology (hypertrophy and synovitis) as well as joint effusion and the articular surface it adds value to the grading of OA (64–73). Small osteophytes, which may be missed on plain radiographs, can be visualized within the joint, allowing for earlier detection of joint pathology. To the authors' knowledge, although it has been proposed as a useful tool in grading human OA (67), this is the first example of MSK US contributing to the grading of OA in dogs.

The majority of dogs had OA in multiple joints in more than one limb and there was a high prevalence of LSD. Indeed, this study is the first of its kind to evaluate treatment protocols encompassing multifocal MSD involving joints, soft tissue support structures and the spine. The most frequently treated joint was the shoulder, followed by hips then elbows with carpi, stifles and hocks less so. Labrador retrievers were over-represented in the study population, accounting for 32% of cases. Consequently, the high prevalence of hip and elbow developmental disease in this breed may have influenced the incidence of joints treated. Elbow dysplasia with secondary OA was also a common reason for presentation in this breed, with previous arthroscopic intervention being a regular feature. In these cases, clinical experience has shown that more aggressive treatment with RM was required and even then, the duration of effect appeared shorter than in patients without prior surgical intervention (AA personal communication). Accordingly, all dogs with elbow dysplasia that had previous arthroscopic surgery received a repeat treatment of MSCs at 12 weeks as a matter of routine.

It is interesting that the joint most often treated was the shoulder. Since intra-articular injections of MSCs and PRP will not affect structures outside the joint capsule, targeted ultrasound guided injection to extracapsular structures was undertaken, with the pathology first identified by MSK US. Consequently, shoulder treatments comprised intra-articular injection and/or ultrasound guided treatment of the shoulder tendons (commonly supraspinatus and biceps tendons), with tendon treatment predominating, indicating a very high prevalence of shoulder tendinopathies. This concurs with a recent study which reported that 55% of dogs with elbow developmental disease had concurrent shoulder tendinopathies (10). Where healing of a tendinopathy was incomplete by 12 weeks, a second treatment of MSCs was injected into the remaining lesions. This was most commonly required in the case of supraspinatus tendinopathy due to the severity, chronicity, and impermeable nature of the tendon enthesis where injection of therapeutics requires a fenestration technique. This, combined with areas of fibrosis and mineralization, limits the volume of MSCs and PRP that can be infused and increases the likelihood of a second treatment being required. However, resolution of mineralization of the supraspinatus tendon and restoration of a normal enthesis after one or two treatments, was an example of the regenerative capacity of MSC therapy in combination with PRP and LT.

According to the Position Statement published by Guest et al. (53), veterinary publications involving MSCs should describe the tissue source, preparation method, cultural method, passage number and method of banking the cells as well as the type of cells, antigenicity, cell dose, dosing schedule, delivery vehicle and method of delivery. The MSCs used here were autologous adipose derived MSCs, culture expanded using standardized protocols, release criteria and strict quality control in a cell culture laboratory authorized by the Veterinary Medicines Directorate, thus complying with the Position Statement (53). Additionally, subsequent recommendations by Ivanovska et al. in the manufacturing of MSCs for the treatment of OA in canine patients were also met (61). MSCs differentiated into chondrocytes and osteocytes but not into adipocytes, despite being derived from adipose tissue. However, according to Sasaki et al. (73) canine MSCs do not always differentiate into adipocytes when induced by a medium optimized for human MSC adipocyte differentiation and therefore this finding is not unusual (73–75). Whereas the efficacy of MSCs was initially considered to be due to their ability to differentiate into musculoskeletal lineages such as chondrocytes and osteocytes, more recently their immunomodulatory and paracrine actions, which influence the inflammatory environment through the release of growth factors and cytokines, are thought to be of more importance (25, 75–77). Despite this lack of clarity regarding the definitive mechanism of action of MSCs, this study has shown that dogs with severe, unresponsive MSD were substantially and rapidly improved by the RM protocol used and that this was sustained for up to 2 years in some dogs. The initial response to treatment can be attributed to the anti-inflammatory effects of the MSC and platelet secretome, but later effects of remodeling, healing and formation of new tissues occur over a more prolonged timeframe. Given that there is evidence that MSCs can persist at the site of injection for more than 10 weeks in OA joints (78) and more than 24 weeks in tendons (79), this could explain the extended sustained improvement following the initial anti-inflammatory effect.

This study looked at treatment responses in naturally occurring disease processes and provides impactful information regarding standardization of canine biological cell products and protocols. This is important for veterinary treatments and also confirms the potential for translational applications in human medicine. Both Ivanovska et al. and Webb et al. have advocated the use of naturally occurring canine disease, treated with standardized RM protocols, as an important area of research to bridge the gap between *in vitro* studies and human clinical trials (61, 80).

For all intra-articular and tendon treatments, MSC injections were accompanied by PRP and followed by a program of laser treatment. This protocol was designed to optimize the efficacy of the MSCs since they respond positively to growth factors released by platelets, and LT *via* PBM (81, 82). The PRP used here was optimal for anti-inflammatory treatments because it contained low numbers of neutrophils and erythrocytes and high concentrations of platelets compared with whole blood (62). Red blood cells and neutrophils have been shown to be deleterious in intra-articular environments through the production of pro-inflammatory mediators and causing synovite death (83). The mean platelet concentration in the prepared PRP was 6.3 times the whole blood concentration, but since the RM injections were a 1:1 mix of MSC suspension and PRP, the overall platelet concentration was approximately 3 times the physiological whole blood platelet concentration. This concentration is in line with the platelet doses reported to be most effective in human clinical studies and animal model studies (84–86). The injection of MSCs with PRP has been shown to be clinically effective in a range of inflammatory diseases (22, 28, 87). Growth factors released by platelets attract and stimulate MSCs to proliferate and to initiate wound healing responses (88). The authors suggest that these actions, together with the formation of a fibrin clot which acts as a scaffold for the MSCs within the injured, inflamed tissues, promoted the healing evident in the joints, and tendons reported in this study.

In order to treat LSD it was considered that MSCs should be injected without the addition of PRP to enable the cells to migrate to all areas of pathology (89). This migratory process, in theory, could be limited by fibrin clot formation and chemotactic cytokines produced by platelets directing MSCs to persist in the epidural space, thereby limiting more widespread effects in the LS region. MSCs have a potent anti-inflammatory effect but, more important in the case of LS stenosis, is their ability to reverse fibrosis, undo nerve compression and alleviate neuropathic pain (90, 91).

Illien-Junger et al. demonstrated homing of human bone marrow derived MSCs into degenerated bovine discs and a subsequent increase in proteoglycan synthesis within the disc demonstrating their migratory as well as regenerative capacity in intervertebral disc disease (IVDD) (92). In a review paper by Oehme et al. (93) four studies in dogs with experimentally induced lumbar IVDD treated with stem cell and progenitor cell transplantation demonstrated positive effects on intervertebral discs (93–99). While experimentally induced disease may differ markedly from that which occurs naturally, these studies are encouraging and support the clinical application described in this study.

Epidural implantation of MSCs at the LS junction provided a repeatable location for placement, allowing local migration in the posterior lumbar region. No adverse reactions were recorded in the clinical record for initial or subsequent treatments. This safety profile, combined with accessibility of the LS junction, makes repeat therapy and long-term management of LSD realistic. This is the first study to report significant improvement in pressure algometry readings together with a very large Cohen's effect size (d) and it demonstrates that MSC epidural injection is a safe, minimally invasive and effective treatment for LSD. In contrast, Salmelin et al. report an incidence of 8.6% of side effects in 150 dogs treated with epidural steroids for LSD (100). Such medical management is not fully effective in controlling LS pain and furthermore surgical patients can develop clinical signs and pain following initial improvements (101).

In dogs with spinal disease such as intervertebral disc disease (IVDD) or spondylosis in the thoracic, cervical, and cranial lumbar regions, IV injection of MSCs was included in the protocol along with epidural MSCs. Stem cells administered intravenously can potentially reach structures where direct implantation is not possible or where epidural administration other than at the LS junction carries the risk of iatrogenic spinal cord injury. In a rodent model, IV stem cells have been shown to migrate to areas of spinal cord damage with positive therapeutic effects (94), including the treatment of neuropathic pain (91). Furthermore, IV MSC administration has been shown to be safe, even in high numbers (2.5 x 10^8^ cells/kg body weight), in people and mammals (95). However, further study is required to evaluate the effects of combining epidural and IV MSCs and their potential synergistic effects on reducing spinal and neuropathic pain.

Laser therapy, an integral part of the treatment protocol, has been used extensively to treat soft tissue and orthopedic injury and pain in animals but there is little information to support its application in conjunction with MSCs (102). *In vitro* studies have shown LT to have beneficial effects on stem cell proliferation (103) and have also indicated that preconditioning of stem cells with photobiomodulation (PBM) can increase cell function, leading to improved healing of wounds (104) and bone (105). Amaroli et al. have reviewed the effects of PBM on MSCs and have shown multiple examples of positive effects relating to their anti-inflammatory action, viability and proliferation and changing the cells lineage differentiation and secretome (106).

Experimental studies carried out *in vivo* have shown that combining PBM with stem cell therapy leads to improved outcomes and that, contrary to commonly held belief, there is no need for a time lapse between stem cell treatment and laser therapy (107–109). While these experimental studies have demonstrated the beneficial effects of combination therapy on soft tissue healing, the use of Class IV LT used simultaneously with injection of culture expanded canine MSCs in naturally occurring MSD has not been previously described, making this study the first to use this combined protocol. However, since all dogs received MSCs and PRP as well as LT any positive effects attributable to LT could not be defined. Nevertheless, Alves et al., using the same LT dose and time points as our study, demonstrated a positive effect on hip joint ROM and pain in dogs with OA, but found mean maximal increases in hip ROM of 8° compared to 14° described in our study (110). Upchurch et al. measured changes in hip ROM following treatment with MSC (stromal vascular fraction) and PRP alone and found a maximal mean increase in ROM of 11° in the 6 months following treatment (111). Although these studies are not directly comparable they suggest that the superior improvements in Hip ROM seen in our study could be due to a synergistic effect of combining MSCs, PRP, and LT but further study is required to substantiate this.

Published evidence to support electrotherapies and physical therapies in combination with RM is lacking and accordingly their use, apart from LT, was restricted in the initial treatment. Where dogs were receiving physical therapies at referral, these were discontinued at the time of diagnosis and fat harvest with physiotherapy or hydrotherapy resumed after the 12-week check demonstrated sufficient healing. For patients receiving a second RM treatment at 12 weeks, physical therapies were not resumed until 18 weeks after initial treatment. Therefore, the significant improvements in objective measures before 12 weeks post-treatment cannot be associated with physical therapies.

The outcome measures used in this study comprised part of patient evaluation aiding in the diagnosis of MSD and measuring clinical change. The information gained was used to inform further treatments where pain and functional parameters deteriorated following the initial treatment.

Although canine gait analysis using force plate data has been used extensively in studies involving orthopedic surgery outcomes, several variables such as walking velocity, head position, position of the handler, and changes in bodyweight between repeat measurements can affect kinematic and force plate data in normal dogs (112, 113). These can influence the reliability of these data and are compounded when a patient has multiple limb gait abnormalities as was the case in our study. Conversely, while the clinical relevance of static limb offloading has not been reported to the same extent, Clough et al., reported good sensitivity and specificity for the detection of both orthopedic disease and objective lameness, and suggested that stance analysis is clinically valuable for measuring response to treatment (29). Additionally, it has been shown that there was no difference in the sensitivity of ground reaction forces and static body weight distribution for measuring hip joint pain and evaluating limb use following treatment (114), hence our decision to use stance analysis in this study.

Stance analysis determines weight distribution whilst the dog is standing on a pressure plate, and it will offload a painful limb and redistribute its weight as a compensatory mechanism. Accordingly, any musculoskeletal pathology will be reflected in the stance making stance analysis an ideal measure of the global effects of MSD. However, there is little published evidence using stance analysis data when reporting treatment effects using RM. Skangals found beneficial, but not statistically significant changes, in weight distribution and joint ROM in 10 dogs with unilateral elbow OA treated with a single intra-articular injection of allogenic MSCs (115). The study found significant improvements in subjective owner reported outcome measures but not for the objective measures which is in marked contrast to the data reported here. Because patients had several limbs involved, often with multiple joint and spinal involvement, comparing affected to non-affected limbs was not possible. Instead, a measure of the absolute departure from a normal weight distribution was calculated. The improvement and normalization of weight distribution following treatment with RM was extremely rapid (from 6 weeks) and maintained to 104 weeks. The authors believe that the improvement resulted from a reduction in pain, with consequent decrease in offloading. Indeed, the fact that the improvement in stance correlated with the decrease in vet pain scores, as shown in Figure 11, lends weight to that hypothesis. A feature of this study was the reporting of clinical as well as statistical significance. In the case of VetMetrica (50) this was demonstrated when the MID of 7 was achieved in E/E, H/C and A/C at time points between 13 and 78 weeks. The demonstration of a significant relationship between change in stance and change in HRQL indicated that the change in stance was also clinically significant.

The ROM of treated joints was measured before and after intra-articular treatments with MSCs and PRP. Normal ROM has been published for a small number of breeds (30, 116–118) but a standard canine range of flexion and extension angles is not available. Nevertheless, following treatment, the increased ROM in this study was often dramatic with many joints regaining what was considered to be a clinically normal ROM by author AA. In the hip and shoulder joints all post-treatment measurements were statistically significantly higher than pre-treatment levels. The same was true for the carpi and elbows except for the 104-week time window. This shows that the ROM in the carpi and elbows starts to reduce approximately 2 years after initial treatment. In the stifle and hock unilateral disease was more common resulting in low sample numbers, and the authors consider that this may have contributed to the lack of statistically significant change in these joints. Also, total ROM measurements were averaged between contralateral paired joints at each time point resulting in a reduced average ROM reading in cases of unilateral treatment. Pre-treatment goniometry recordings were taken whilst the animal was under GA, negating the effect of pain limiting extension or flexion, but post-treatment angles were recorded in the conscious patient. Since Clark et al. found that the total ROM in the elbow increased by up to 11 degrees under sedation or GA compared with when the dog was conscious (119), the post-treatment results reported in this study may be an underestimation of the actual increase in total ROM. Nevertheless, the reported increases in total ROM were significant in most treated joints. These findings support the regenerative potential of MSCs since, for a joint to gain a greater total ROM, there must be some remodeling of the osteoarthritic new bone, changes in joint capsule thickness and elasticity or muscle-tendon length, which is restricting either flexion or extension.

A recent study investigated joint ROM in 20 working police dogs with hip OA before and after PBM therapy (110). This prospective, positive-controlled, double-blinded study showed that PBMT reduced pain levels and improved clinical findings in dogs with hip OA compared to the NSAID meloxicam for up to 90 days post-treatment (110). The increased total ROM in the treated group was less than that found in the present study following treatment with MSCs, PRP, and PBM therapy. Although the studies are not directly comparable and differ in timing of PBM treatments the therapeutic levels of LT (10 j/cm^2^) were identical. This provides evidence to suggest that the improvements in hip total ROM are greater when MSCs and PRP are combined with PBM. Carr et al. found a mean increase in elbow ROM of 6° in dogs with elbow OA treated with PRP alone at 90 days post treatment compared with a 2° increase in the control group in a small cohort of Labradors (120). This is considerably less than the 22° increase in elbow ROM observed in a much larger number of dogs with elbow OA in this study suggesting that combining MSCs and LT with PRP has a greater effect on increasing ROM than PRP alone.

Pressure algometry has been used with success in numerus human studies (121–123), but this is the first study to use the technique to objectively measure changes in LS pain thresholds in dogs in a clinical setting. Over 80% of the treated dogs received injections of MSCs into the epidural space at the LS junction for the treatment of lower back pain associated with LSD in addition to concurrent appendicular pathologies. The prevalence of LSD suggests that changes in gait and weight distribution caused by appendicular MSD may predispose to accelerated degeneration at the LS junction, secondary to altered biodynamics of the spine. The PA results showed a statistically significant increase in pain threshold between pre-treatment measurements and all subsequent measurements up to 104 weeks following epidural injection of MSCs and this was supported by a significant reduction in analgesic and anti-inflammatory medication usage following treatment. The decrease in analgesic requirement was statistically significant up until 32 weeks on average, thus highlighting the pain-relieving properties of the RM treatment. It is noteworthy that the pre-treatment PA measurements were taken when the dogs were receiving analgesic medication whilst many of the post-treatment readings were recorded when analgesic medication had been discontinued or dramatically reduced, so the analgesic effect of MSC injection at the LS junction was likely to be underestimated.

The clinical significance of both ROM and PA was demonstrated by calculating the effect size between pre-treatment and the 6 weeks following treatment. Unlike statistical significance, effect size as measured by Cohen's (d) is independent of the sample size and is considered a good indication of clinical significance (124). An effect is considered large if d > 0.8 (125). In this study, Cohen's effect size (d) ranged from 1.05 to 2.14 for ROM and 2.2 for PA which would be considered as large to huge according to Sawilowsky (126).

This study measured LS pain following treatment with RM, however, physical changes to the LS junction were not investigated. It would be interesting to discover what regenerative changes, if any, coincide with the resolution of pain following treatment. Since there would be no requirement for anesthesia, MSK US could be a more economic non-invasive option than MRI to monitor change in hydration of the disc by determining its echogenicity and elastography. This technique has already shown the regenerative capacity of MSCs in tendons in this study by demonstrating reversal of scar tissue, fibrosis, and mineralization, resolution of inflammatory change, complete healing of partial tendon tears and restoration of normal entheses following treatment. Accordingly determining the regenerative potential of MSCs at the LS junction using MSK US will be the focus of further studies.

Many orthopedic specialists prefer objective outcome measures to client-reported outcome measures (CROMs), which include CMIs or HRQL measures, because they believe that the caregiver placebo effects, seen with subjective measures, could compromise the accuracy of their results. However, because CROMs are recognized as important by the FDA and the EMA, these have become an integral part of veterinary clinical trials. Even though Cook stipulated that, in orthopedic trials, CROMs should include a HRQL measure (42), there is still a tendency for researchers to rely on CMIs, perhaps because studies in dogs have shown that objective gait analysis and certain CMIs produce equivalent outcomes (53). It is interesting to note that, in their Position Statement Guest et al. use a low CMI score to indicate a “negligible identification of impact on quality of life” (53). While this inference may be true, it is an indirect association as CMIs are not measures of QOL. While CMIs measure only the functional limitation imposed by the disease, VetMetrica™ HRQL instruments measure the emotional as well as the physical impact of the disease, and furthermore do so on a continuum from the worst to the best QOL (46).

VetMetrica assessments were completed by owners in accordance with the study protocol. In the domain A/C the improvement in all post-treatment time windows compared with pre-treatment was significant, with a similar result in E/E after 6 weeks and in H/C and C/R after 12 weeks. This indicated that the improvement in PWB preceded that of EWB by 6 weeks. This contrasts with treatment of OA with NSAIDs where an improvement in EWB is often seen before that of any improvement in PWB (47).

Currently studies showing a significant relationship between HRQL and objective measures are lacking, but the improvement in HRQL seen here mirrored that seen in all objective outcome measures. Indeed, formal analysis showed that there was a significant relationship between change in all HRQL domain scores and change in weight distribution, as measured by stance analysis, providing evidence of convergent construct validity for VetMetrica™. Of all the objective measures used, stance analysis was considered the most appropriate measure to correlate with HRQL measures since it is a global measure of musculoskeletal function. Similarly, the relationship between change in stance and change in vet assessments mirrored that of the change in stance and change in HRQL scores, with significant relationships for all three subjective measures, thus supporting the hypothesis that subjective and objective measures can produce equivalent outcomes in trials. However, it should be noted that if the pain and QOL impact assessments had been carried out by less experienced clinicians the relationship between these and stance may not have been as robust.

Importantly, in addition to the improvement in HRQL being statistically significant, clinical significance was demonstrated by the fact that the change in scores between pre- and post-treatment was equal to or greater than the MID in E/E, H/C, and A/C. Although this was not the case for C/R, the fact that this domain is affected by the temperament of the dog, may have been a contributing factor.

The veterinary assessments for pain and QOL impact as scored on a 0–10 numerical rating scale (NRS) provided additional subjective outcome measures in this study. The NRS is an ordinal scale and a major drawback to its use is inter-observer variability. However, the fact that a single observer completed all assessments removed that risk. Regarding assessing pain in non-verbal individuals, the FDA Guidance for Industry (127) states that an observer cannot validly attribute a pain score on behalf of another individual but can observe behaviors that indicate pain. Accordingly, in this study the pain assessment question was carefully worded to ensure that the score reflected the amount of pain the observer *felt* the dog was suffering. In this case, the observer (AA), a very experienced clinician dealing only with orthopedic conditions, was well placed to make that judgement. There is an argument that the same would not apply to judging QOL impact, since the clinician does not see the dog in its home environment, but the result of the analysis was remarkably similar with highly significant time window effects (*p* < 0.001) and all post-treatment means were significantly lower than pre-treatment. This may have reflected the close bond between author AA, the dog and its owner, formed over a considerable period and facilitated by an extensive consultation process supplemented by very regular updates on progress.

This study demonstrated a significant reduction in pain following treatment with RM, as measured by PA, stance analysis (offloading due to pain), vet pain scores and a significant reduction in analgesic requirement. Limitations of the study include the lack of a control group but the retrospective nature of the study made the reporting of a control group impossible. Had it been possible, the authors would have considered a control group inappropriate for the following reason: the study population consisted of client-owned dogs with MSD that was unresponsive to standard treatments at the time of referral, so continuing such treatment would be likely to prolong pain and suffering. Nevertheless, each dog was its own control, having been non-responsive to conventional treatments prior to the demonstration of significant improvements following targeted regenerative therapies. The authors consider a further limitation to be the fact that each objective outcome measure group size was different. This was due to the broad spectrum of pathologies present within the study population and omissions from the clinical record, however all dogs had at least two outcome measures recorded. Similarly, the number of HRQL assessments was reduced because the difference between these and the other outcome measurements had to be within 14 days of each other to be included in the analysis. However, since there were 954 assessments from 212 dogs up to week 104 this number was considered adequate for the study. Lack of blinding of author AA could be considered a limitation of the study in view of evidence of caregiver bias (128). This would be a serious consideration in a RCT but is not reported as such in RWD studies where data is collected from various sources including electronic patient records, as was the case in this study (129).

## 5. Conclusion

This study presents results of a longitudinal study being carried out in a veterinary practice specializing in RM, for the treatment of a large population of dogs with naturally occurring MSD that was unresponsive to conventional treatment. Importantly, the study complied with the minimal criteria for reporting MSCs in orthopedic applications published recently by Guest et al. (53). This, together with the use of several different validated outcome measures by a single, experienced, veterinary practitioner make this a uniquely important study of the efficacy of RM in a clinical setting. Results have shown that using a standardized protocol of known numbers of autologous adipose derived MSCs, combined with leukocyte poor PRP where appropriate, injected into all areas of MSK pathology, resulted in rapid and profound positive effects in the patient's pain status (pressure algometry, vet score), function (stance analysis, goniometry) and QOL (VetMetrica™ HRQL assessment tool) for up to 2 years. Furthermore, this study endorses the use of VetMetrica™ in canine MSD by demonstrating a significant positive relationship with an objective measure of MSK function, providing evidence to support its value in orthopedic clinical trials.

The authors believe that this large scale, comprehensive study adds considerably to the evidence required to support the use of MSCs in canine orthopedic conditions and lays the foundations for further research regarding their regenerative potential. Furthermore, RM should be considered an important component in the multimodal approach to treatment of chronic degenerative MSK conditions in dogs, although care in selecting the most scientifically robust MSC and PRP products is imperative.

## Data availability statement

The original contributions presented in the study are included in the article/Supplementary material, further inquiries can be directed to the corresponding author.

## Ethics statement

The animal study was reviewed and approved by RCVS Ethics Review Panel. Written informed consent was obtained from the owners for the participation of their animals in this study.

## Author contributions

AA, JM, and JR: conceptualization. TS: statistical analysis. All authors contributed to the article and approved the submitted version.

## Footnotes

^1^Greenside Veterinary Practice, Greenside Farm, St Boswells, UK.

^2^Cell Therapy Sciences Ltd, Coventry, UK.
