# Supplementary material for: Efficacy of autologous mesenchymal stromal cell treatment for chronic degenerative musculoskeletal conditions in dogs: A retrospective study
Source: Front Vet Sci. 2023 Jan 13;9:1014687. doi: 10.3389/fvets.2022.1014687 (PMC9880336; doi:10.3389/fvets.2022.1014687)
Supplement: Supplementary file 2 [file Table_2.DOCX]

**Cell Therapy Product Batch Release Criteria**

All MSC treatments achieved the following batch release criteria:

1. **Microbiological Testing**
2. Final product microbial testing using 100µl of the final product injected into a Bloodgrow bottle and subsequently seeded onto a blood agar plate was negative after 4 days incubation. If the final product microbial test was positive, then the product was not released.
3. **Cell Morphology Check**
4. Photographs of the cell culture taken using the x10 objective lens should be taken of the same field daily (excepting weekends) and cell morphology compared with standard cell culture photographs. An experienced cell culture biologist certified that the cell morphology of the culture was within normal bounds. Unacceptable morphology included:
5. Large round cells with the appearance of “Fried Eggs” making up more than 1/50 of adherent cells.
6. Rounding up and detaching cells make up more than 1/20 of the adherent cells.
7. Excessively piled up cells typical of a super confluent culture
8. **Cell Count and Viability**
9. The number of cells in the final product vial was not less than 2.5 million cells per ml.
10. The viability of the cells in the final product vial after cryopreservation and rapid thawing was >85% viable as estimated by the Trypan Blue exclusion method.

Each product batch was provided with a Certificate of Analysis in which the above criteria were listed and results quoted. The Certificate of Analysis was be signed by the Laboratory Manager before the product was released for dispatch.
