# Supplementary material for: Efficacy of autologous mesenchymal stromal cell treatment for chronic degenerative musculoskeletal conditions in dogs: A retrospective study
Source: Front Vet Sci. 2023 Jan 13;9:1014687. doi: 10.3389/fvets.2022.1014687 (PMC9880336; doi:10.3389/fvets.2022.1014687)
Supplement: Supplementary file 4 [file Table_4.DOCX]

**Date of Consultation: Hospital Name: Your name: Dog Name:**

**Do you consider the dog to be in perfect health?** **Y N**

**Please place an X in the appropriate column to assess the following conditions:**

| None Mild Moderate Severe End Stage |
| --- |
| Degenerative Joint Disease |
| Obesity |
| Painful cancer |
| Non-painful cancer |
| Chronic skin disease |
| Chronic medical condition |
| Cardiac disease |
| Neurological disease |
| Chronic ear disease |
| Chronic dental disease |
| Other |

**If you selected other, please specify:**

**On a scale of 0 to 10 with 0 being no impact and 10 being the most impact, please assess how much the dog’s health status is reducing its quality of life (QoL):**

**No impact 0 1 2 3 4 5 6 7 8 9 10 Impact could not be greater**

**On a scale of 0 to 10 with 0 being no pain and 10 being the pain could not be worse, please indicate what amount of pain you feel the dog is suffering?**

**No pain 0 1 2 3 4 5 6 7 8 9 10 Pain could not be worse**

**What treatment are you prescribing for this dog?**

**How has the dog’s general health status changed since the previous consultation?**

| **Much Worse** | **Worse** | **Unchanged** | **Better** | **Much Better** |
| --- | --- | --- | --- | --- |

**Are you recommending euthanasia at this consultation yes/no**
