# Supplementary material for: Efficacy of autologous mesenchymal stromal cell treatment for chronic degenerative musculoskeletal conditions in dogs: A retrospective study
Source: Front Vet Sci. 2023 Jan 13;9:1014687. doi: 10.3389/fvets.2022.1014687 (PMC9880336; doi:10.3389/fvets.2022.1014687)
Supplement: Supplementary file 6 [file Table_6.DOCX]

Numbers of records (numbers of dogs) for each analysis

|  |  |  |  |  | ROM | | | | | |
| --- | --- | --- | --- | --- | --- | --- | --- | --- | --- | --- |
| Time window | Vetmetrica QoL – 4 domains | Vet QoL & Vet pain score | Stance score | LS pain | Carp | Elb | Sho | Hoc | Sti | Hip |
| Pre-implant | 183 (174) | 207 (188) | 192 (184) | 124 (121) | 36 (35) | 116 (109) | 164 (154) | 22 (22) | 49 (46) | 129 (120) |
| 0-6 weeks | 125 (102) | 85 (81) | 91 (90) | 63 (63) | 18(18) | 41 (41) | 64 (64) | 12 (12) | 19 (19) | 43 (43) |
| 7-12 | 113 (98) | 118 (105) | 134 (124) | 99 (90) | 22 (20) | 72 (68) | 105 (97) | 11 (10) | 23 (22) | 67 (62) |
| 13-18 | 95 (90) | 113 (109) | 109 (106) | 86 (83) | 22 (20) | 70 (69) | 101 (99) | 10 (9) | 27 (26) | 78 (74) |
| 19-24 | 60 (55) | 69 (65) | 64 (64) | 40 (40) | 11 (11) | 33 (33) | 49 (49) | 8 (8) | 10 (10) | 32 (32) |
| 25-48 | 124 (83) | 112 (85) | 95 (77) | 55 (48) | 12 (9) | 50 (40) | 72 (57) | 10 (9) | 17 (13) | 47 (41) |
| 49-78 | 134 (85) | 108 (79) | 85 (63) | 48 (33) | 10 (8) | 37 (28) | 59 (43) | 3 (3) | 18 (12) | 46 (36) |
| 79-104 | 120 (68) | 94 (59) | 85 (55) | 53 (36) | 23 (14) | 47 (32) | 69 (45) | 10 (6) | 19 (14) | 53 (36) |
|  |  |  |  |  |  |  |  |  |  |  |
|  | 954 (212) | 906 (223) | 855 (228) | 568 (171) | 154 (51) | 466 (123) | 683 (191) | 86 (28) | 182 (60) | 495 (158) |

Fitted means from linear mixed models and letter codes from Tukey multiple comparisons; means within a column not sharing a letter in common were significantly different (p<0.05).

| Time window | Energetic/ Enthusiastic | | Happy/ Content | | Active/ Comfortable | | Calm/ Relaxed | | Vet QoL | | Vet pain score | | Stance | |
| --- | --- | --- | --- | --- | --- | --- | --- | --- | --- | --- | --- | --- | --- | --- |
| Pre-implant | 34.56 | c | 38.69 | c | 28.35 | d | 41.79 | c | 5.85 | a | 5.74 | a | 16.77 | a |
| 0-6 weeks | 36.22 | bc | 40.62 | bc | 33.11 | c | 42.37 | bc | 2.31 | bc | 1.59 | cd | 6.69 | b |
| 7-12 | 40.15 | ab | 42.98 | abc | 34.78 | abc | 43.94 | abc | 2.02 | c | 1.30 | d | 5.96 | b |
| 13-18 | 42.64 | a | 44.17 | ab | 36.54 | abc | 46.46 | ab | 1.85 | c | 1.37 | cd | 5.88 | b |
| 19-24 | 45.86 | a | 48.80 | a | 40.25 | a | 48.42 | a | 1.84 | c | 1.30 | cd | 5.16 | b |
| 25-48 | 43.85 | a | 47.12 | a | 36.54 | abc | 46.52 | ab | 2.53 | bc | 2.01 | cd | 7.70 | b |
| 49-78 | 43.72 | a | 46.76 | a | 38.24 | ab | 47.06 | a | 2.52 | bc | 2.03 | bc | 6.86 | b |
| 79-104 | 40.62 | ab | 43.75 | ab | 33.92 | bc | 46.53 | ab | 3.04 | b | 2.79 | b | 8.28 | b |
|  |  |  |  |  |  |  |  |  |  |  |  |  |  |  |
|  |  |  |  |  |  |  |  |  |  |  |  |  |  |  |
|  | Shoulder ROM | | Hip ROM | | Elbow ROM | | Stifle ROM | | Carpi ROM | | Hock ROM | | LS pain | |
| Pre-implant | 102.61 | b | 104.12 | b | 105.52 | c | 119.76 | b | 146.82 | c | 119.14 | a | 6.89 | b |
| 0-6 | 111.37 | a | 118.09 | a | 127.99 | ab | 128.34 | ab | 158.61 | ab | 133.88 | a | 11.50 | a |
| 7-12 | 112.52 | a | 115.57 | a | 127.38 | a | 125.95 | ab | 162.75 | ab | 121.53 | a | 12.38 | a |
| 13-18 | 114.02 | a | 118.61 | a | 126.22 | ab | 131.17 | a | 166.45 | a | 128.46 | a | 12.17 | a |
| 19-24 | 112.19 | a | 117.89 | a | 126.50 | ab | 124.10 | ab | 162.05 | ab | 126.00 | a | 12.72 | a |
| 25-48 | 112.69 | a | 115.95 | a | 122.61 | ab | 124.50 | ab | 164.40 | ab | 137.27 | a | 12.28 | a |
| 49-78 | 113.92 | a | 116.95 | a | 124.39 | ab | 127.24 | ab | 166.25 | ab | 116.83 | a | 12.57 | a |
| 79-104 | 113.79 | a | 117.09 | a | 115.35 | bc | 131.12 | a | 154.92 | bc | 120.74 | a | 11.68 | a |
