# Supplementary material for: Efficacy of autologous mesenchymal stromal cell treatment for chronic degenerative musculoskeletal conditions in dogs: A retrospective study
Source: Front Vet Sci. 2023 Jan 13;9:1014687. doi: 10.3389/fvets.2022.1014687 (PMC9880336; doi:10.3389/fvets.2022.1014687)
Supplement: Supplementary file 7 [file Table_7.DOCX]

**Details of the specific Musculoskeletal Disorders treated in the study population.**

| **MSD** | **Site of pathology** |
| --- | --- |
| **Osteoarthritis** | Metacarpal phalangeal joints  Carpi  Elbows  Shoulders  Hocks  Stifles  Hips |
| **Tendinopathies** | Supraspinatus, Biceps  Subscapularis  Pollicis longus  Iliopsoas  Achilles  Gluteal. |
| **Desmopathies** | Cranial cruciate ligament  Medial glenohumeral ligament  Joint collateral ligaments. |
| **Spinal Conditions** | Lumbosacral disease  Intervertebral Disk Disease  Spondylosis  Dorsal articular facet OA |
| **Specific MSD** | Hip dysplasia  Elbow developmental diseases/Elbow dysplasia  Osteochondrosis  Gracilis contracture  Medial shoulder syndrome/instability  Femoral neuritis  Immune Mediated Polyarthritis |
