# Supplementary material for: Efficacy of autologous mesenchymal stromal cell treatment for chronic degenerative musculoskeletal conditions in dogs: A retrospective study
Source: Front Vet Sci. 2023 Jan 13;9:1014687. doi: 10.3389/fvets.2022.1014687 (PMC9880336; doi:10.3389/fvets.2022.1014687)
Supplement: Supplementary file 8 [file Table_8.DOCX]

Median (interquartile range) for HRQL domains Energetic/Enthusiastic (E/E), Happy/Content (H/C), Active/Comfortable (A/C) and Calm/Relaxed (C/R) at pre-treatment and all subsequent time windows, with the increase in medians at all post- treatment time windows from pre-treatment values shown. An increase of 7 and above represents a clinically significant change.

| **Domain** |  | **Pre-treatment (PT)** | **0-6w** | **7-12w** | **13-18w** | **19-24w** | **25-48w** | **49-78w** | **79-104w** |
| --- | --- | --- | --- | --- | --- | --- | --- | --- | --- |
| **E/E** | **Median (IQR)** | 36.4(21.5) | 38.6(19.7) | 41.0(17.3) | 46.6(18.6) | 47.6(15.7) | 44.7(13.9) | 46.4(17.3) | 41.0(20.1) |
|  | **Increase in median from PT** |  | 2.2 | 4.6 | 10.2 | 11.2 | 8.3 | 10 | 4.6 |
|  | | | | | | | | | |
| **H/C** | **Median (IQR)** | 36.8(18.8) | 39.3(23.7) | 42.8(22.5) | 44.2(22.5) | 50.4(18.3) | 48.2(17.8) | 49.0(21.5) | 41.6(22.4) |
|  | **Increase in median from PT** |  | 2.5 | 6 | 7.4 | 13.6 | 11.4 | 12.2 | 4.8 |
|  | | | | | | | | | |
| **A/C** | **Median (IQR)** | 26.0(10.4) | 31.2(12.0) | 32.0(13.2) | 34.6(14.1) | 34.9(25.1) | 35.0(12.6) | 36.0(18.1) | 29.7(13.2) |
|  | **Increase in median from PT** |  | 5.2 | 6 | 8.6 | 8.9 | 9 | 10 | 3.7 |
|  | | | | | | | | | |
| **C/R** | **Median (IQR)** | 42.2(13.4) | 43.2(15.8) | 43.7(14.8) | 45.9(16.1) | 50.0(13.2) | 46.3(13.0) | 48.3(16.9) | 46.8(13.6) |
|  | **Increase in median from PT** |  | 1.0 | 1.5 | 3.7 | 7.8 | 4.1 | 6.1 | 4.6 |
